# Evaluation of genetic structure in European wheat cultivars and advanced breeding lines using high-density genotyping-by-sequencing approach

**DOI:** 10.1186/s12864-020-07351-x

**Published:** 2021-01-28

**Authors:** Mirosław Tyrka, Monika Mokrzycka, Beata Bakera, Dorota Tyrka, Magdalena Szeliga, Stefan Stojałowski, Przemysław Matysik, Michał Rokicki, Monika Rakoczy-Trojanowska, Paweł Krajewski

**Affiliations:** 1 Rzeszow University of Technology, Powstańców Warszawy 12, 35-959 Rzeszów, Poland; 2 Institute of Plant Genetics, Polish Academy of Science, Strzeszyńska 34, 60-479 Poznań, Poland; 3 Warsaw University of Life Sciences, Nowoursynowska 166, 02-787 Warszawa, Poland; 4 West Pomeranian University of Technology Szczecin, Słowackiego 17, 71-434 Szczecin, Poland; 5 Plant Breeding Strzelce Group IHAR Ltd., Kasztanowa 5, 63-004 Tulce, Poland; 6 Poznań Plant Breeding Ltd., Główna 20, 99-307 Strzelce, Poland

**Keywords:** Genetic variation, Breeding, Single nucleotide polymorphisms, Population structure, *Triticum aestivum* L

## Abstract

**Background:**

The genetic diversity and gene pool characteristics must be clarified for efficient genome-wide association studies, genomic selection, and hybrid breeding. The aim of this study was to evaluate the genetic structure of 509 wheat accessions representing registered varieties and advanced breeding lines via the high-density genotyping-by-sequencing approach.

**Results:**

More than 30% of 13,499 SNP markers representing 2162 clusters were mapped to genes, whereas 22.50% of 26,369 silicoDArT markers overlapped with coding sequences and were linked in 3527 blocks. Regarding hexaploidy, perfect sequence matches following BLAST searches were not sufficient for the unequivocal mapping to unique loci. Moreover, allelic variations in homeologous loci interfered with heterozygosity calculations for some markers. Analyses of the major genetic changes over the last 27 years revealed the selection pressure on orthologs of the gibberellin biosynthesis-related *GA2* gene and the senescence-associated *SAG12* gene. A core collection representing the wheat population was generated for preserving germplasm and optimizing breeding programs.

**Conclusions:**

Our results confirmed considerable differences among wheat subgenomes A, B and D, with D characterized by the lowest diversity but the highest LD. They revealed genomic regions that have been targeted by breeding.

**Supplementary Information:**

The online version contains supplementary material available at 10.1186/s12864-020-07351-x.

## Background

Common wheat (*Triticum aestivum* L.), which is an important cereal crop grown worldwide on 220 million ha, accounts for 20% of the total calories consumed by the global population. In Europe, wheat is cultivated on 62 million ha, including 2.3 million ha in Poland [1]. Various approaches are currently being used to increase wheat yields to satisfy the expected demand for food sources. Doubling the wheat yield by 2050 [2] is a challenging goal and will require the application of the increased genetic diversity of landraces well adapted to different stresses [3], synthetic wheat varieties [4], and wild relatives [2]. One of the milestones toward the development of high-yielding and climate-smart ‘next generation varieties’ was the sequencing of the 17 Gb allohexaploid wheat (AABBDD) genome [5, 6]. The wheat reference sequence was annotated with various genetic markers that were historically used for evaluating genetic resources to enhance wheat production.

The genetic diversity of breeding materials is critical for increasing wheat nutritional quality, yield, and yield stability. Evaluating the extent of the genetic diversity among adapted, elite germplasm may be useful for estimating the genetic variability among segregating progeny [7]. Elite varieties are recurrently used for the subsequent breeding aimed at accumulating the optimal combination of alleles. Thus, genetic variability may decrease, which may hinder efforts to further increase the yield potential of wheat varieties.

Although hybrid breeding may be a viable option for increasing wheat yields, it requires technological advances that can modulate floral development and architecture to enable outcrossing, the regulation of male sterility, and fertility restoration [8, 9]. Previous studies revealed that hybrids may increase yields by 10% across diverse environments and improve the yield stability [10, 11]. Various strategies have been developed for hybrid wheat production [9, 12], including chemically induced male sterility [13], seed production technology [9], and the application of the tight linkage between the dominant dwarfism gene *Rht-D1c* and *Ms2* [12]. The *Ms1* and *Ms2* genes, which were recently sequenced, are useful for the large-scale, low-cost production of male-sterile female lines necessary for hybrid wheat seed production [9, 12, 14]. Among the various hybridization systems available for producing hybrid cultivar seeds, the most promising seems to involve cytoplasmic male sterility (CMS), which is based on the interaction between nuclear and mitochondrial genes, and has been widely used for breeding various crops [15]. Irrespective of the final system used for hybrid seed production, the components should represent separate gene pools to ensure good combining ability. Information related to the genetic diversity among adapted lines helps breeders select suitable parents for hybridizations that maximize heterosis and combine useful genes in an adapted genetic background [16].

Different marker systems have been employed to study the genetic diversity of wheat and to generate information useful for wheat breeding and improvement in national and international programs. Genotyping methods that evolved from various types of PCR and hybridization-based markers as well as methods for detecting single nucleotide polymorphisms (SNP) have exploited microarray genotyping platforms and genotyping-by-sequencing (GBS). The genetic diversity in wheat accessions was previously assessed with single-locus markers, including simple sequence repeats (SSR), or competitive allele-specific PCR (KASP) [17–23].

On the basis of sample barcoding, next-generation sequencing technology was adapted for the simultaneous discovery of SNPs and presence–absence variations (PAV) in multiple genotypes. Additionally, the application of GBS technologies (e.g., DArTseq) is considered to be the most cost-efficient method [24] for genomics-based breeding [25–27]. Different collections of wheat landraces have been genotyped based on GBS [28], Illumina 9 K and 90 K SNP arrays [29, 30], DArTseq [3, 31], exome capture [32], Illumina GoldenGate [33], and the 35 K Axiom WhtBrd-1 Array [34]. The high map density obtained with SNP markers is particularly useful for assessing gene pool variations and marker–trait associations as well as for genomic selection, determining population structures, and QTL mapping [35–38]. It is also relevant for accurately selecting accessions for a core collection, which is a limited set of accessions representing the genetic diversity of a crop species and its wild relatives, with minimal repetitiveness [39–42].

The mining of genetic diversity in modern cultivars adapted to local climatic conditions is a continuous process [20], and is a prerequisite for discerning pools of genotypes and diverse parents for effective breeding programs and the subsequent production of hybrid seeds. In the present study, 509 European wheat cultivars and advanced breeding lines (Table S1) were examined regarding their genetic diversity and population structure. The objectives of this study were to: a) assess the genetic diversity in pre-breeding programs involving modern genotypes from Europe and advanced breeding lines; b) compare the distribution of SNPs among wheat chromosomes; c) generate genotyping data for a genome-wide association study (GWAS); and d) define a core collection representative of the European gene pool currently used for breeding.

## Results

### Marker mapping and selection

**Fig. 1:**
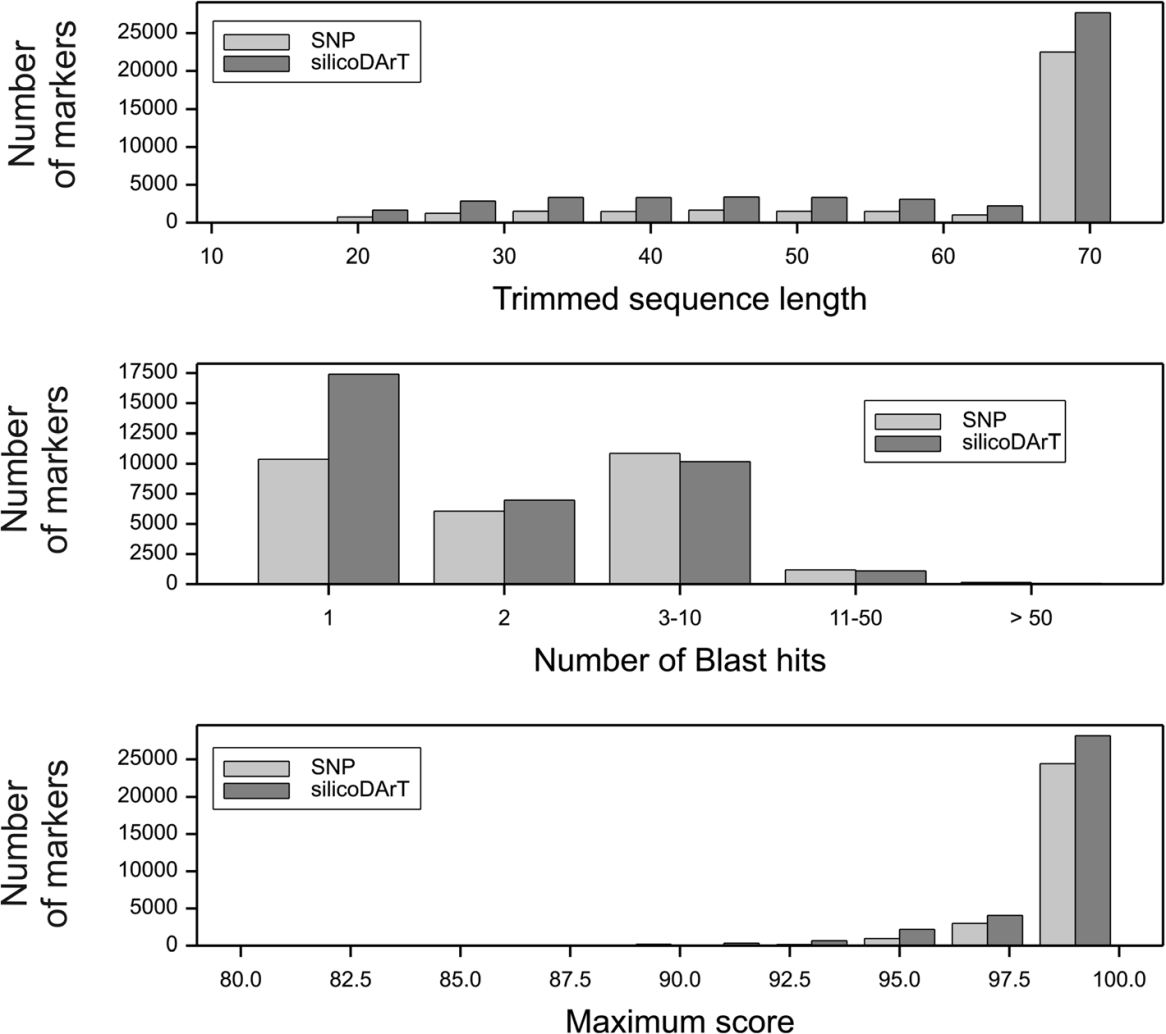
Distributions of trimmed sequence length, number of BLAST hits, and maximum BLAST scores for SNP (gray) and silicoDArT (dark gray) markers

Raw SNP and silicoDArT datasets contained 33,135 and 50,929 markers, respectively (Table 1). The mean trimmed sequence used for mapping to the reference genome was longer for SNP markers (Table 1). The fraction of marker sequences mapped to the reference genome (under the given BLAST threshold criteria) was greater for SNPs (86.4%) than for silicoDArTs (70.1%). However, the mapping quality assessed according to the number of BLAST hits per marker and the maximum similarity score was lower for SNPs (Table 1, Fig. 1). Additionally, 86.3 and 88.9% of the SNP and silicoDArT markers were mapped uniquely (i.e., the maximum score was recorded for a single location), respectively. A comparative analysis of the distribution of trimmed sequences classified by the sequence length and maximum BLAST score indicated that most of the SNP and silicoDArT markers between 20 and 50 bp had a maximum score below 95%, which corresponded to decreased specificity.

**Table 1:** Marker dataset characteristics and differences in distributions (Mann-Whitney rank test)

Marker type	Number of markers				Trimmed sequence length: mean, range (nt)	Maximum score per marker, range
	total	mapped in reference genome		selected (% of total)		
		mapped (% of total)	mapped uniquely (% of mapped)			
SNP	33,135	28,615 (86.4%)	24,691 (86.3%)	13,499 (40.7%)	60.79, 15–69	85.0–100
silicoDArT	50,929	35,719 (70.1%)	31,770 (88.9%)	26,369 (51.8%)	57.20, 15–69	83.3–100
					*p* < 0.001	*p* = 0.036

Significance level for difference between SNP and silicoDArT

Only uniquely mapped markers were selected for additional analyses. For filtering, the “MVF > 0.1” criterion was applied to both marker sets, whereas the “call rate > 0.6” criterion was applied only to SNP markers. Regarding the silicoDArTs, the minimum call rate was 0.76. Following the filtering, 13,499 (40.7%) of the SNP markers and 26,369 (51.8%) of the silicoDArT markers were retained.

### Characteristics of filtered datasets

**Fig. 2:**
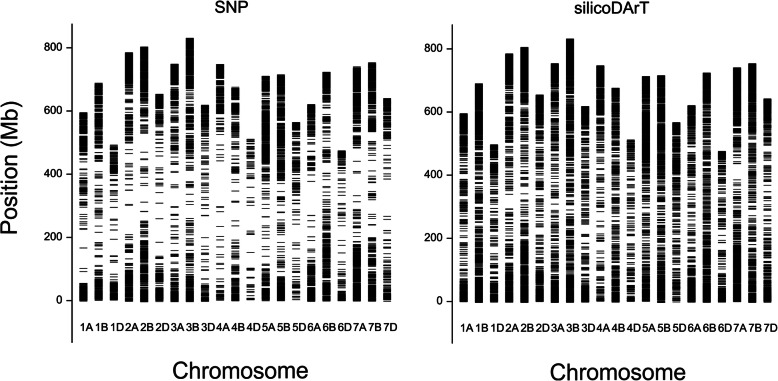
Physical mapping of 13,499 SNP and 26,369 silicoDArT markers on wheat chromosomes 1A - 7D

The physical locations of 13,499 SNP and 26,369 silicoDArT markers (Table 1) on wheat chromosomes (Fig. 2, Table S2) indicate that they were not homogeneously distributed among chromosomes, with distal chromosomal fragments covered more than internal, pericentromeric regions. However, silicoDArT markers were more equally distributed than the SNPs, and the median distance between markers was more that 2-times greater for SNP markers (171 kb) than for silicoDArT markers (67 kb). The median distances between SNP markers were 140, 220, and 420 kb in subgenomes A, B, and D, respectively. The corresponding distances between silicoDArT markers were 66, 87, and 187 kb. Chromosomes from homeologous group 2 and chromosome 4D most often had the lowest and highest median distances between markers, respectively (Table S2). The highest quality markers mapped at a single position, with a score of 100, constituted 25.7 and 38.8% of the SNP and silicoDArT markers, respectively (Table S3).

**Fig. 3:**
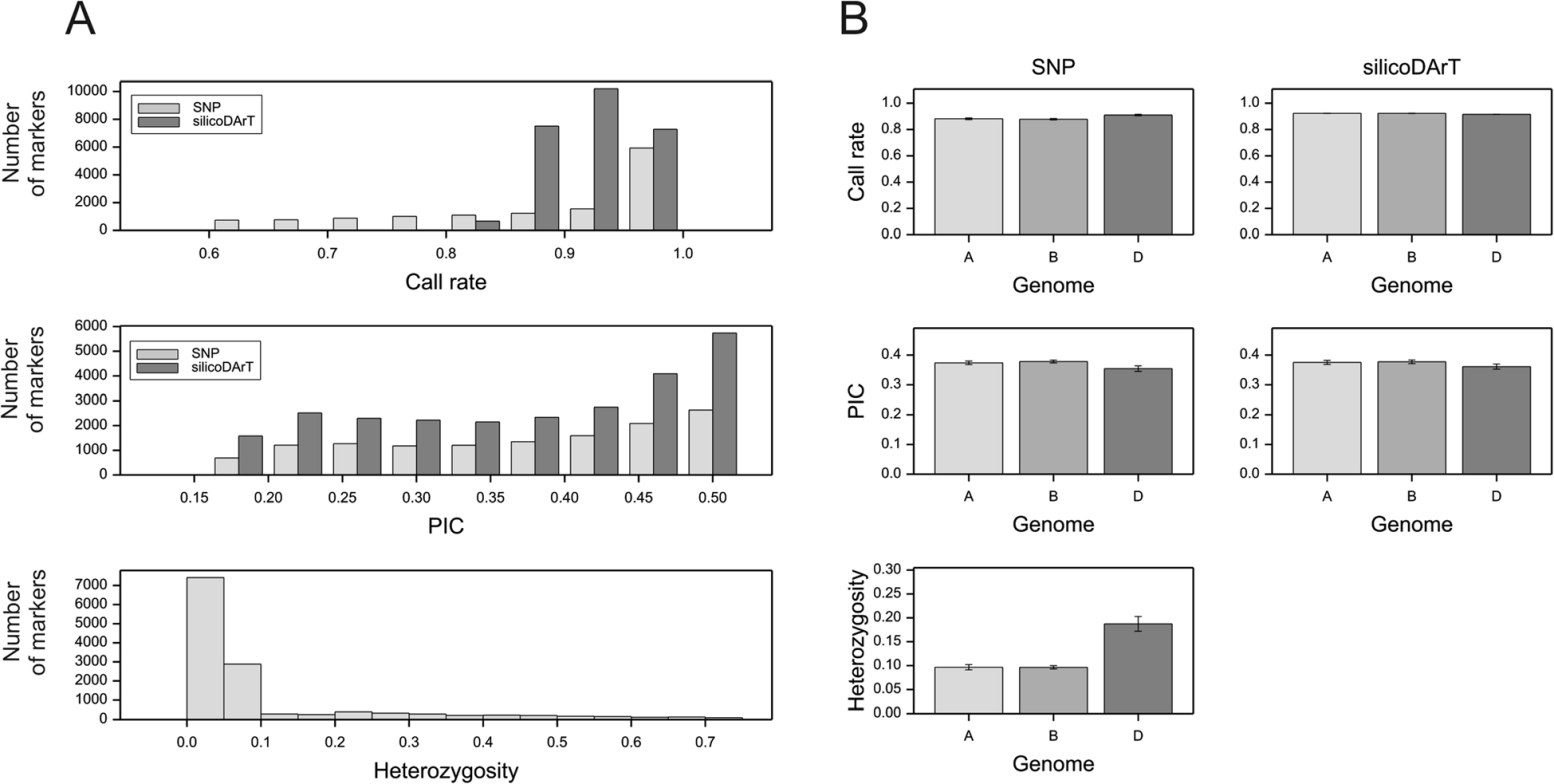
Overall distribution of SNP (gray) and silicoDArT (dark gray) marker characteristics (**a**) and their subgenome specificity (**b**) characteristics

The distributions of call rates for SNPs and silicoDArTs (Fig. 3a) indicate that the minimum call rate was lower for SNPs, but the mode of its distribution was higher (0.99) than that for silicoDArTs (0.97). The average call rate for SNPs was significantly (*p* < 0.001) higher in subgenome D (0.91) than in subgenomes A or B (0.88, Fig. 3b). No accession was removed from the analysis because of a high fraction of missing genotypic data. The distributions of PIC values for SNP and silicoDArT markers were similar. Additionally, the mean PIC values for both SNPs and silicoDArTs were significantly higher in subgenomes A and B (0.37–0.38) than in subgenome D (0.35–0.36, *p* < 0.001; Fig. 3b). The PIC values were especially low for chromosome 3D (Fig. S1A). The heterozygosity of the SNP markers did not exceed 0.75, with 10,310 markers exhibiting a heterozygosity of less than 0.1 (Fig. 3a). Moreover, heterozygosity was not equally distributed among wheat subgenomes. Specifically, compared with subgenomes A and B, the heterozygosity (0.19) was 2-times higher in subgenome D (Fig. 3b), especially in chromosome 4D (Fig. S1A).

**Fig. 4:**
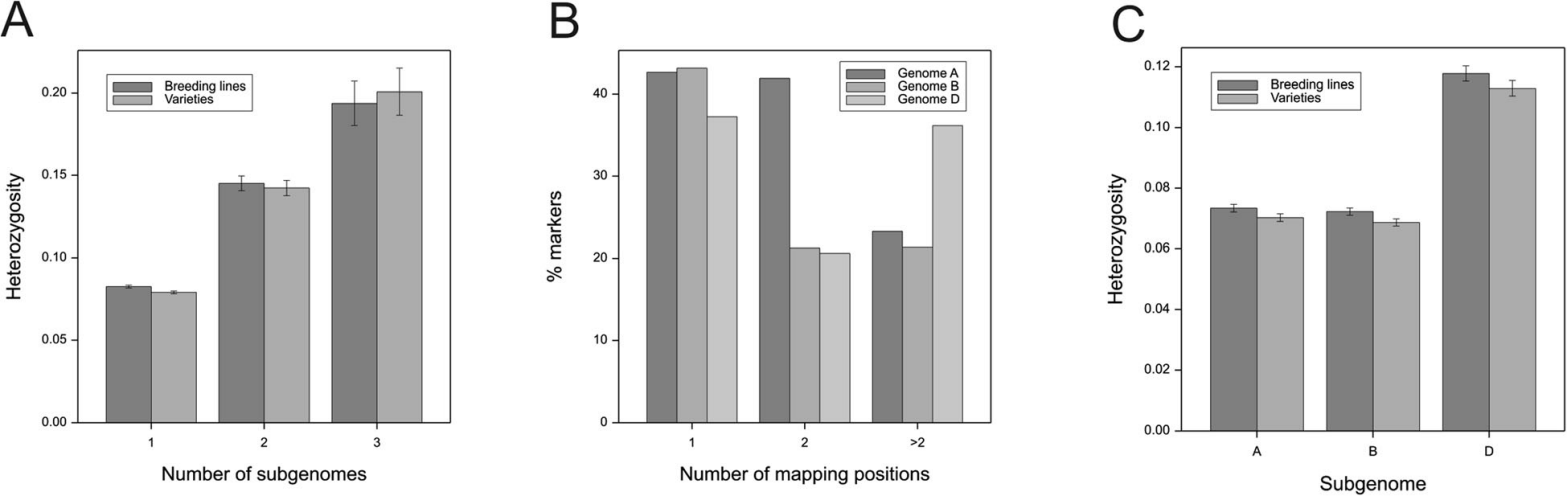
Mean heterozygosity of SNP markers mapped simultaneously to one, two, or three subgenomes (**a**). Fractions of SNPs with a single best hit in subgenomes A, B, or D and with 1, 2, or > 2 mapping positions (**b**). Heterozygosity of unique (one best hit) SNP markers in varieties and lines mapped to wheat subgenomes A, B, and D (**c**)

Additional analyses were performed to clarify the increased heterozygosity of the markers in subgenome D. By analyzing the raw marker data (i.e., before selection), we determined that the heterozygosity of hemizygous markers was as high as 0.19–0.20 (Fig. 4a). Further analyses of the total number of hits for the sequences with one best hit indicated that the SNPs from subgenome D (ascribed based on the best hit) were mapped more frequently in alternative loci than the SNPs from subgenomes A or B (chi-square test, *p* < 0.001, Fig. 4b). For all subgenomes, the heterozygosity of markers in the breeding lines was slightly higher than that in the cultivars (Fig. 4c).

### Linkage disequilibrium

**Fig. 5:**
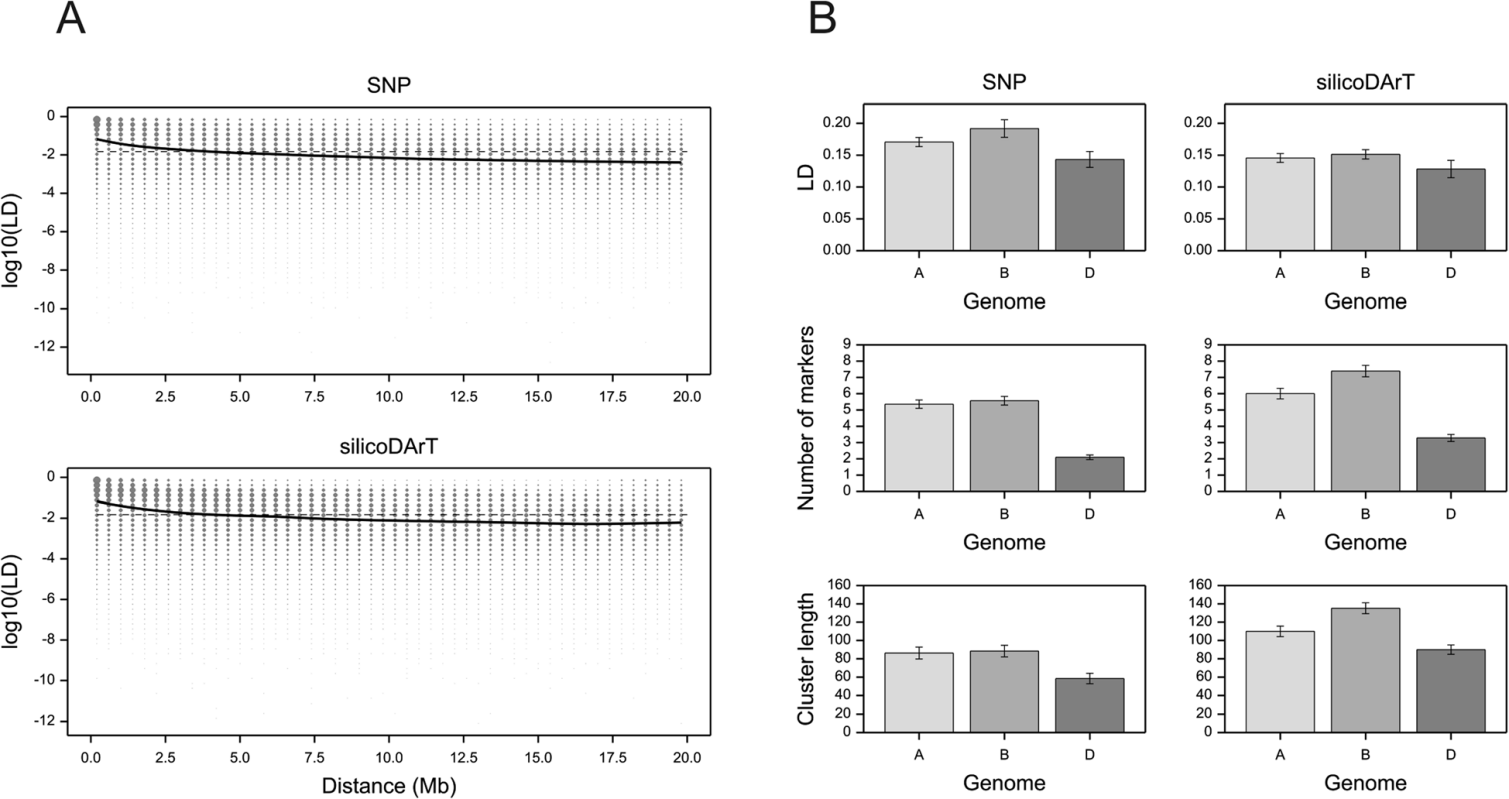
Plots of LD vs physical distance between markers, with 0–20 Mb distance intervals (**a**). The dashed line marks the 95th percentile of LD for unlinked markers computed for random pairs of markers from different chromosomes (0.0157 and 0.0149 for DArTseq and DArT, respectively). The continuous line results from the fitting of a smoothing-spline regression (with 12 df) of LD on distance. Characteristics of LD within subgenomes and of clusters of markers identified based on the LD (**b**)

The relationship between LD values and physical distances between markers is presented in Fig. 5a. For both datasets, the expected LD (estimated by smoothing splines) was greater than the 95th percentile of LD for unlinked markers (random markers from different chromosomes) for pairs of markers located at a distance of up to approximately 5 Mb. Therefore, for wheat genomes, 4.1% of loci collocated in a 5 Mb region are in LD. However, the mean LD in the 5 Mb region based on both marker systems differed among the three wheat subgenomes, and was lowest for subgenome D (Fig. 5b), especially for chromosomes 4D and 6D (Fig. S1B).

The grouping of markers according to the LD (performed to analyze the population structure) resulted in clusters with more markers and longer clusters (in Mb) in subgenomes A and B than in subgenome D (Fig. 5b, Fig. S1B). A total of 2162 and 3527 clusters (i.e., groups of markers assumed to be unlinked) were detected for the SNP and silicoDArT markers, respectively. An example of the SNP marker clusters for chromosome 1A is presented in Fig. S2. Analyses of the LD between intersecting SNP and silicoDArT markers revealed some pairs with a low LD resulting from non-unique mapping or genotyping errors.

### Annotation of markers

Of 13,499 SNP markers, 4389 (32.51%) were located in genes. Of 26,369 silicoDArT markers, 5934 (22.50%) had trimmed sequences that overlapped with coding sequences. The frequencies of transitions (A > G, G > A, C > T, and T > C) and transversions (other variants) among SNPs were 63.17 and 36.83%, respectively. There were significantly more transitions in subgenome A (64.64%) than in subgenome D (61.08%) (Pearson chi-square test, *p* = 0.013). A prediction of the effects of 3060 SNPs (23.27%) located in protein-coding regions uncovered 33 (1.08%) variants with “HIGH” effects, 1493 (48.79%) with “LOW” (synonymous) effects, and 1534 (50.13%) with “MODERATE” (nonsynonymous) effects. The corresponding frequencies of divisions between subgenomes A, B, and D are listed in Table S4. The SNPs with LOW or MODERATE effects were more frequent in subgenome D than in subgenomes A or B, whereas the intergenic and intron variants (MODIFIERS) were less frequent.

The computed kinship matrices were processed via a PCoA, and the relationship between the polymorphism of SNP markers and the variability represented by PCO1 and PCO2 was assessed by ANOVA. The computed F-statistic values are visualized for SNPs located in coding sequences (with predicted HIGH, LOW, or MODERATE coding effects) in Fig. S3. The SNPs most related to PCO1 were located predominantly in regions 2A: 702,956,966–726,296,256 (four SNPs), 2B: 666,654,689–719,453,838 (32 SNPs), and 2D: 563,009,137–595,508,041 (10 SNPs). The SNPs related to PCO2 were mainly in regions 3A: 692,987,178–734,790,501 (three SNPs), 3D: 597,923,720–615,474,140 (nine SNPs), and 4A: 713,605,603–742,585,853 (26 SNPs). There were no SNPs with HIGH effects in these regions. The GO annotation and overrepresentation analysis of the 48 genes harboring SNPs related to PCO1 revealed several overrepresented processes (i.e., response to auxin stimulus, response to hormone stimulus, response to endogenous stimulus, and response to organic substance) (genes: TraesCS2D02G494600, TraesCS2B02G522500, TraesCS2A02G494300, and TraesCS2B02G522200). There were no overrepresented GO terms among the 55 genes harboring SNPs related to PCO2.

The three SNPs with the largest F-statistic values for PCO1 were identified in homeologous genes TraesCS2A02G463000, TraesCS2B02G484700, and TraesCS2D02G463600 located on chromosomes 2A, 2B, and 2D, respectively, according to the best hit method. However, the presence of six allelic variants in three SNPs located in a 53 bp marker sequence resulted in five haplotypes. High heterozygosity (0.61%) in chromosome 2A and 2D loci was identified because the same allelic variants overlapped between subgenomes, and in fact exhibited a hemizygous nature (Table S5). This example indicates that regarding hexaploidy, exact matches between sequences in BLAST analyses are not sufficient for the unequivocal mapping to unique loci.

### Population structure

**Fig. 6:**
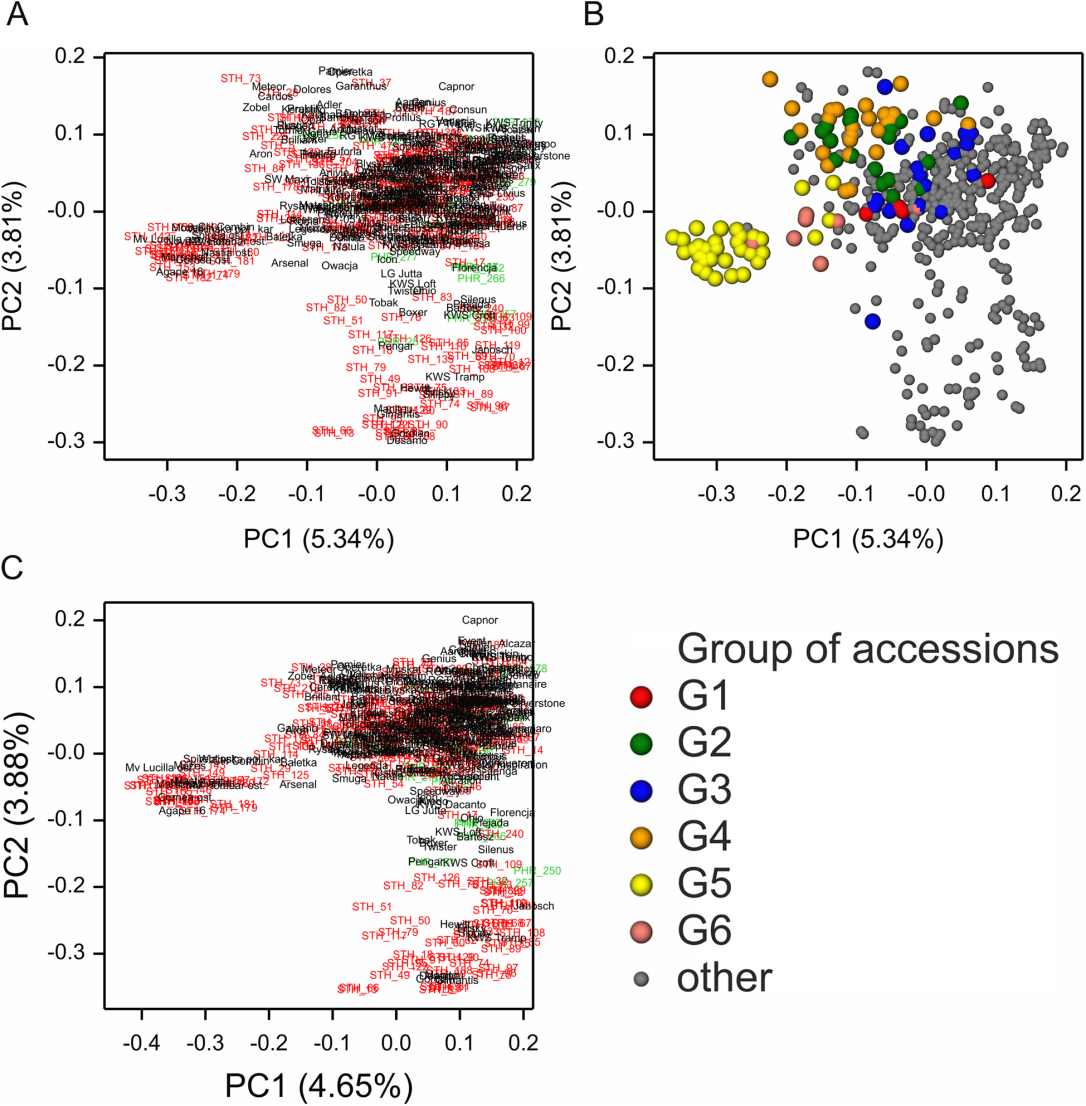
Visualization of the population structure revealed via principal coordinate analysis of kinship matrices for SNP and silicoDArT data. In the graph on the right, accessions belonging to groups classified as stable in the bootstrap analysis are marked by large colored circles

**Fig. 7:**
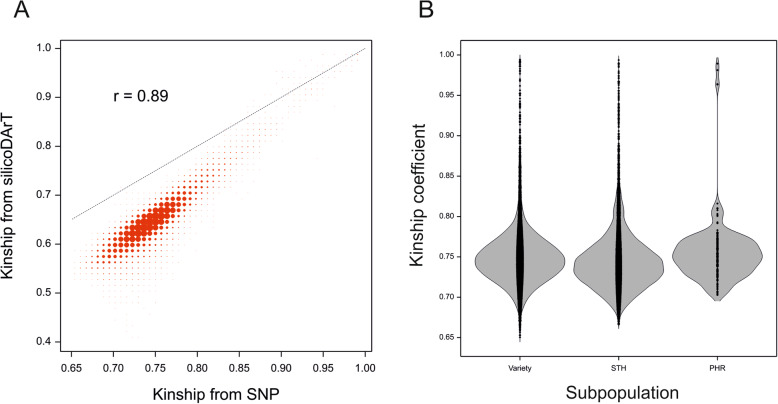
Comparison of the distributions of kinship coefficients derived from SNP and silicoDArT data (**a**) and within groups of accessions (**b**)

The population structure visualized by a PCoA of the kinship (coancestry coefficients) matrix of accessions derived from SNP and silicoDArT markers revealed similar features (Fig. 6). A bootstrap analysis uncovered six stable groups comprising 112 accessions and 397 genotypes that were not grouped. The largest and most distinct group was group no. 5, which included 12 varieties and 24 STH accessions, all originating from eastern (Ukraine and Belarus), central (Hungary), and parts of southern Europe (Table S1). The kinship coefficients based on SNP and silicoDArT data were highly correlated (*r* = 0.89), but the silicoDArT coefficients were lower (Fig. 7a). The distribution of kinship coefficients revealed a higher mean internal kinship within varieties (0.75) and PHR accessions (0.76) than within STH accessions (0.74) (Mann-Whitney U test, *p* < 0.01; Fig. 7b).

To identify major genetic changes over the last 27 years, the available information regarding the year of first registration for 263 varieties (Table S1) was treated as a quantitative trait and used for a GWAS (Fig. S4). The six most significant markers (Benjamini-Hochberg corrected *p* value < 0.002) associated with the registration year were identified from a set of 13,499 SNP markers (Table 2). Two of the genes with polymorphisms related to the registration year were orthologs of the *GA2* gene involved in gibberellin biosynthesis, and a third gene was identified as an ortholog of the *SAG12* senescence-associated gene of *Arabidopsis thaliana*. A clear difference between the oldest (1992–1998) and newest (2018–2019) varieties was observed regarding the genotype at these six loci (Table 3). Functional KASP markers have been proposed for four out of six SNPs related to the registration year (Table S6). High GC content and high hairpin stability prevented from conversion of 1134008|F|0–29|CA and 1237275|F|0–14|AG SNPs, respectively, into KASP markers.

**Table 2:** Six SNP markers with polymorphisms associated with the year of first registration for varieties

Marker ID	Chromosome	Position	Corrected *p* value	Frequency REF	Frequency ALT	Gene ID	Translation effect	GO annotation	*A. thaliana* ortholog
1134008|F|0–29|CA	2A	6,420,426	0.001445	0,42	0,58	–	–	–	–
997149|F|0–32|TC	2A	8,142,744	0.001036	0,40	0,60	TraesCS2A02G017300	MODIFIER	–	–
1002630|F|0–15|GA	2D	12,332,115	0.001036	0,73	0,27	TraesCS2D02G030100	LOW	GO:0016829 lyase activity GO:0000287 magnesium ion binding GO:0010333 terpene synthase activity GO:0046872 metal ion binding GO:0000287 magnesium ion binding GO:0010333 terpene synthase activity GO:0016102 diterpenoid biosynthetic process	GA2
1204154|F|0–9|CT	Un	12,441,675	0.001036	0,47	0,53	TraesCSU02G008700	LOW	GO:0016829 lyase activity GO:0000287 magnesium ion binding GO:0010333 terpene synthase activity GO:0009686 gibberellin biosynthetic process GO:0009570 chloroplast stroma GO:0046872 metal ion binding GO:0009899 ent-kaurene synthase activity GO:0000287 magnesium ion binding GO:0010333 terpene synthase activity GO:0016102 diterpenoid biosynthetic process	GA2
1015908|F|0–35|GC	Un	236,255,714	0.001036	0,30	0,70	TraesCSU02G163600	MODIFIER	GO:0008234 cysteine-type peptidase activity GO:0006508 proteolysis	SAG12
1237275|F|0–14|AG	Un	248,331,142	0.001464	0,59	0,41	TraesCSU02G169100	LOW	GO:0016788 hydrolase activity, acting on ester bonds	–

**Table 3:** Genotypes of the oldest and newest varieties at six loci associated with the registration year

Variety	Registration year	Marker ID					
		1134008|F|0–29|CA	997149|F|0–32|TC	1002630|F|0–15|GA	1204154|F|0–9|CT	1015908|F|0–35|GC	1237275|F|0–14|AG
Kobra Plus	1992	C/C	C/C	G/A	T/T	G/C	A/A
Roma	1992	C/A	C/C	G/A	T/T	G/C	A/A
Rysa	1998	C/C	C/C	G/A	T/T	G/C	A/A
Plejada	2018	−/−	T/T	G/G	C/C	C/C	G/G
Euforia	2018	A/A	T/T	G/G	C/C	C/C	G/G
RGT Treffer	2018	−/−	T/T	G/G	C/C	C/C	G/G
SY Orofino	2018	−/−	T/T	G/G	C/C	C/C	−/−
Comandor	2018	A/A	T/T	G/G	C/C	C/C	G/G
Venecja	2019	−/−	T/T	G/G	C/C	C/C	G/G

### Core collection

**Fig. 8:**
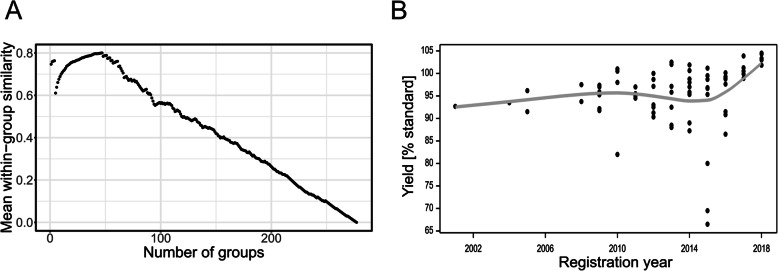
Plot of the average within-group similarity relative to the number of groups (**a**). Scatterplot depicting the relationship between yield of varieties and their registration year (based on the data from 2015 to 2018 post-registration trials; regression line drawn using smoothing spline function with 4 degrees of freedom) (**b**)

To create the core collection for a wheat variety subpopulation, we split the varieties into 1, 2, …, 277 clusters via kinship-based hierarchical clustering. For each partition, we calculated the average within-group kinship values assuming that a single-element group has a similarity equal to 0. The maximum average kinship value obtained for 47 clusters was considered to be the optimal number of clusters (Fig. 8a). A core collection was formed by taking one entry from each cluster, resulting in a collection comprising 47 accessions (approximately 17% of the whole collection).

To select the representative variety from each cluster, we analyzed the yield data for winter wheat varieties generated in post-registration trials performed in years 2015–2018 at two levels of protection, A1 and A2 (Table S7; data obtained from Research Center for Cultivar Testing COBORU [43]). Among the wheat varieties tested in these trials, 75 varieties were from the HYBRE collection. The first level of protection, A1, corresponds to common agricultural practices, whereas the second one, A2, corresponds to intense agricultural practices (e.g., increased nitrogen fertilization, foliar multi-component preparations, and protection against lodging and diseases). Accordingly, we formed two core collections corresponding to both agricultural practices. From each cluster, we selected the accession with the highest mean yield or a random accession if yield data were unavailable for the group. The results are presented in dendrograms (Fig. S5) and as a list of the selected accessions (Fig. S6A). In both core collections, the representative variety was chosen based on the yield data for 30 clusters. Additionally, 29 selections were common to A1 and A2. In one case, the difference between the data for A1 and A2 was due to cultivars Florus and Franz, with mean yields differing in A1 by 0.33% (of the yield of standards) and in A2 by − 1.33%. Differences between the distributions of kinship coefficients in the whole collection and in the two core collections were not significant (chi-square test, *p* = 0.298 and 0.303, Fig. S6B).

We also used yield data from post-registration trials 2015–2018 to assess the differences of yield potential between the old and the new varieties (Fig. 8b). The increasing trend is disturbed by the presence of some relatively low-yielding varieties, especially those registered in the years 2010 and 2015.

## Discussion

### Marker mapping and selection

In this study, GBS technology (DArTseq) was applied to evaluate the genetic diversity and population structure of 509 wheat accessions. This system represents a cost-effective alternative to gene-based array platforms [24, 44]. Previous studies demonstrated that arrays adapted for high-throughput genotyping of bread wheat offer resolutions ranging from 8 K to 550 K [29, 45–51].

The DArT system enables the detection of two types of markers, namely SNPs and silicoDArT markers representing PAVs. In the panel of 509 wheat accessions, we selected 37,868 markers comprising 13,499 (40.7% of all identified) and 26,369 (51.8% of all identified) non-redundant, high quality SNPs and PAVs, respectively. The shift to PAV markers in the SNP method may be due to the sensitivity of the applied restriction enzymes (PstI, HpaII, and HhaI) to cytosine methylation and the destruction of some fragments by excess TaqI endonuclease. In the newly synthesized allopolyploid wheat, alterations in methylation patterns affected about 13% of a random set of genomic loci [52]. As a consequence of filtering, our SNP map was less dense and less specific than the PAV map.

For wheat, which has a large and complex genome, an analysis of the LD decay enabling the evaluation of marker density is especially important for high-quality association mapping and marker-assisted selection [53–55]. The map density is considered to be sufficient for these purposes if the distance between markers is less than the LD decay [36]. In the current study, the LD for the SNP and silicoDArT markers decayed for about 5 Mb and the mean distance between markers ranged from 66 kB (silicoDArTs in subgenome A) to 420 kB (SNPs in subgenome D), indicating the map density was sufficient. The 5 Mb LD decay means that 3400 equally dispersed, non-redundant markers should be sufficient to scan 17 Gb of the wheat genome. Nevertheless, 13,499 and 26,369 SNPs and silicoDArTs were selected, respectively.

### Differences between subgenomes

The markers were unequally distributed among three subgenomes, with fewer markers in subgenome D (16.1 and 19.7% of the SNPs and silicoDArTs, respectively). An uneven distribution of markers among wheat subgenomes A, B, and D is a phenomenon that has been previously reported. For example, in several studies, a smaller proportion of markers was mapped to the “youngest” subgenome (i.e., subgenome D) than to subgenomes A and B [22, 29, 56, 57]. An analysis of the whole-genome resequencing data for eight wheat lines identified 3.3 million SNPs, with 41% located in subgenome A, 49% in subgenome B, and 10% in subgenome D [48]. A very similar marker distribution in homoeologous genomes (40% in subgenome A, 48% in subgenome B, and 12% in subgenome D) was determined with the 280 K Affymetrix Axiom SNP array [51] as well as for 2114 wheat genes (41, 43, and 16% in subgenomes A, B, and D, respectively) [58]. As suggested by Würschum et al. [59], these observations were because the array was biased regarding polymorphic SNPs from different genomes.

Relatively high variability in diversity among wheat chromosomes and uneven diversity patterns along large chromosomal segments may result from the synergy of genetic drift and selection under limited gene flow, self-pollination, and low effective recombination [46, 58, 60, 61]. The presence of structural rearrangements may also shape chromosome-specific changes in genetic diversity. An examination of evenly distributed KASP markers resulted in the detection of 44 types of translocations in 42 of 58 wheat nested association mapping populations. An earlier investigation uncovered more translocations on the chromosomes of subgenome B than on the subgenome A chromosomes [22]. This is consistent with the finding that the proportions of loci in deletion-bin maps that are incongruent with the linkage map locations are higher for subgenomes A and B (10.8 and 12.4%, respectively) than for subgenome D (8.8%) [58]. In the current study, the calculated median distances between silicoDArT markers were 66.4, 87.2, and 187.2 kb in subgenomes A, B, and D, respectively, which corresponded with the low saturation of chromosomes 4D and 4B, thereby confirming the results described above.

The PIC values differed among subgenomes, with the lowest values for subgenome D, for both the SNP and silicoDArT markers mapped to the subgenome. This may have resulted from the relatively few markers mapped to subgenome D and their low polymorphism due to the targeted selection of this subgenome. Rosyara et al. [92] suggested that the current bread wheat subgenome D has limited genetic diversity resulting from a few hybridization events involving *Aegilops tauschii* during hexaploid wheat genome evolution, limited gene flow from *Ae. tauschii* to bread wheat, and the intensive human selection of bread wheat, which further decreased the diversity. The same relationships among the PIC values for SNP markers mapped to wheat subgenomes A, B, and D revealed in this study were detected in other studies, including those by Chao et al. [62], Lopes et al. [63], Liu et al. [64], and Eltaher et al. [57] as well as in a study by Mir et al. [65] regarding SSRs.

The heterozygosity of SNP markers in the studied population did not exceed 0.75. Moreover, the heterozygosity of nearly a third of the markers was lower than 0.1. Similar values, expected for self-pollinating hexaploid wheat, were reported by other researchers [48, 57, 66, 67]. The heterozygosity was not equally distributed among three subgenomes, and substantially higher values were calculated for subgenome D (2-fold higher than the values for subgenomes A and B), especially for chromosome 4D. Similarly, Liu et al. [55] described the heterozygosity differences in subgenomes A, B, and D among four populations (Chinese landraces, modern Chinese cultivars, Pakistani landraces, and modern Pakistani cultivars), but the highest heterozygosity of markers in subgenome D was observed only for modern Chinese cultivars. In contrast, Bhatta et al. [66] did not detect any significant differences among subgenomes A, B, and D in terms of marker heterozygosity, both in bred and synthetic hexaploid wheat lines. A case study involving three markers presented herein (Table S5) suggest that increased heterozygosity is generally due to the fixation of different alleles in homoeologous genomes, resulting in hemizygosity. During mapping, marker sequences were compared with the reference genome sequence of the primitive ‘Chinese Spring’ variety. Therefore, the shift in the heterozygous markers annotated with the best hit method to subgenome D suggests that most of the primitive alleles in modern wheat varieties and lines are from subgenome D rather than from subgenomes A and B.

In the present study, subgenome D was characterized by the lowest LD, especially for silicoDArT markers on chromosomes 4D and 6D, which, except for chromosome 1D, corresponded to marker saturation. Subgenome D reportedly exhibits high LD despite having the fewest mapped markers [29, 60, 68, 69]. Thus, many traits are inherited together in blocks (for a review, see [61]). In an earlier study by Wang et al. [46], the LD decayed 2- to 3-times more slowly in subgenome D than in subgenomes A and B. Additionally, Akhunov et al. [58] proved that in subgenome D, the average Wall’s B value, which is a measure of LD, is 0.81 and is significantly higher than that in subgenomes A and B, indicative of a greater LD among the subgenome D genes. Furthermore, no significant differences in intra-locus LD were detected among the chromosomes from subgenomes A and B. Chao et al. [60] explained that the greater LD in wheat subgenome D than in subgenomes A and B was due to the recent introgression and population bottleneck accompanying the origin of hexaploid wheat, which does not fully reflect our observations. The low LD in subgenome D in our population may have been a consequence of a relatively high heterozygosity in this subgenome reflecting the actual hemizygous state of some of the markers.

### Annotation

In wheat, the three distinct subgenomes and an interchromosomal gene duplication rate of 20 to 30% strongly influence the annotation of markers [70, 71]. Thus, genotyping is complicated by the presence of homoeologous and paralogous loci [29, 46, 48, 72]. In terms of functional categorizations (GO slim terms), there is no biased gene loss in any of the subgenomes, and functional copies of genes encoding transcription factors have been retained in all three subgenomes [73]. The exclusion of markers targeting homeoloci was proposed to reliably allocate individual haplotypes into their respective genomes [58]. However, some classes of genes involved in energy harvesting, metabolism, and growth may be associated with crop productivity [73]. Moreover, the expression of all homeoalleles encoding the same or similar functional proteins leads to the formation of novel “hybrid” enzymes, resulting in greater physiological versatility and wider adaptability [74]. Therefore, markers based on homeoloci are important for a GWAS and should be accepted after they are annotated based on stringent threshold levels.

In the current study, only 23.1% of the SNPs (3129) were located in coding regions. Although the percentage of markers located within genes encoding proteins was smaller for silicoDArTs than for SNPs (22.50 and 32.51%, respectively), the total number of silicoDArT markers was higher by as much as 1545. Only a few (approximately 1%) of the polymorphic SNPs detected in coding regions were classified as highly affecting protein functions. Most of the polymorphic SNP loci had low or moderate effects. In an earlier study by Wang et al. [46] involving a high-density genotyping array, the percentage of SNPs located in coding regions was 57.78%, which is more than double the percentage determined in our study. This discrepancy resulted from the selection of gene-related sequences for the array. However, stringent locus-specific annotations seem to be easier for GBS technologies than for arrays because a simple cut-off for eliminating markers with mismatches exceeding a single nucleotide can be used. In the current study, the transitions and transversions respectively accounted for 63 and 37% of the SNPs, which were consistent with the corresponding percentages calculated by Wang et al. [46] (72% transitions and 28% transversions), but the proportion of synonymous mutations was lower (48.87% vs 62.54%). Different panels of markers were most likely targeted in these two assays.

### Genetic diversity and population structure

The heterozygosity in the studied population was relatively low, with a mean value of 0.11 and nearly 73% of accessions with a heterozygosity value less than 0.1. Generally, such values are typical for self-pollinated species. However, the previously reported heterozygosity values for hexaploid wheat (i.e., means for the whole populations and individual subpopulations) are slightly higher. In a study by Wang et al. [46], the mean heterozygosity of a population comprising six common wheat subpopulations representing different regions of origin was slightly higher at 0.19, ranging from 0.15 to 0.24 among individual subpopulations. Similar results were obtained by other researchers, including Eltaher et al. [57] and Kumar et al. [75]. The comparison between subpopulations revealed differences. The heterozygosity of advanced breeding lines was higher than that of cultivars. Theoretically, advanced breeding materials should be highly homozygotic. A higher share of heterozygotes may be due to their origin because they were usually derived from interbreeding programs involving unrelated parental components. Beneficial heterozygotic loci can be preferentially selected, leading to their overrepresentation, as suggested by Charlesworth and Willis [76]. The recorded differences may reflect some variability in the breeding strategies applied by the two companies that provided the study materials.

Among six subpopulations, group no. 5 was the most distinct and consisted of accessions from eastern, central, and southern Europe. Phenotypically, they are early, winter cold-hardy forms, with relatively long straw and lower yield potentials, and are well adapted to the continental climate conditions. Despite the weak yield potential, some of the group no. 5 accessions, distinct from most of the contemporary wheat Polish breeding materials, may be valuable resources for agronomically desirable traits.

### Population structure versus variety age

Of 13,499 SNPs included in a GWAS, six markers clearly distinguished the oldest (1992–1998) and newest (2018–2019) varieties. Two of these markers were located in *GA2* genes, whereas a third marker was detected in the *SAG2* gene, with the annotation being based on orthology to *A. thaliana* genes. The *GA2* gene affects the gibberellin biosynthesis pathway by mediating the conversion of ent-copalyl diphosphate to the gibberellin precursor ent-kaur-16-ene [77]. Gibberellins are plant growth-promoting hormones that influence various developmental processes, including stem elongation, lodging tendency, seed germination, floral induction [78], and dormancy via ABA–GA crosstalk [79], ultimately affecting yield [80]. The senescence-associated gene *SAG2*, which encodes a cysteine protease, is responsible for developmental senescence-specific cell death during apoptosis, heavy metal detoxification, and the hypersensitive response [77]. Delays in leaf senescence have recently been reported to impact wheat growth and yield [81]. Because of their multiple functions, both genes were likely unintentionally subjected to selection in the process of breeding for yield improvement. The differences in yielding of the oldest and newest varieties were visible in the studied population, although they were not as large as the differences demonstrated in [82], primarily because we used data from contemporary experiments, whereas in [82] historical data, obtained under different management, were used. Notably, the two groups of old and new varieties included in this study differ inter alia regarding plant height and lodging, with values in the oldest and the newest varieties of 86.7 and 7.6 and 102.2 and 6.5, respectively [83]. Moreover, the oldest varieties were heterozygotes when the newest ones – homozygotes, in one out of two markers located in the GA2 genes (1002630|F|0–15| GA) and the marker located in the SAG2 gene (1015908|F|0–35|GC). Due to application of pooling of plant material for genotyping, this may be the effect of heterogeneity of individuals. However, homozygosity can be of great help when creating markers for MAS.

### Core collection

An important objective of this study was to construct a core collection representative of the structure of the whole collection. Core collections are crucial for gene bank management and they are useful for elucidating the diversity within a population [84]. As described by Odong et al. [42], three types of core collections may be formed. First, the whole collection is represented by the most similar accessions, whereas the second type characterizes the extreme accessions of the whole collection and the third type represents the distribution of the accessions in the original set. The first core collection type is ideally a uniform representation of the original genetic content, unlike the second type, which includes entries that are as diverse as possible, and the third type, which provides an overview of the composition of the whole collection. We decided to generate the third type of core collection. It consisted of 47 accessions, representing approximately 17% of the whole collection. Thus, our collection satisfies the condition set by van Hintum [85] that the core collection should comprise between 5 and 20% of the base collection. Additionally, a comparison of the distributions of kinship coefficients in the whole collection and in the core collections confirmed that our results satisfy the requirements for the third core collection type described by Odong et al. [42]. The final step for creating a core collection was performed based on the available yield data. The two versions of the core collection corresponding to two agricultural practices were very similar, implying that the genotype × environment interaction minimally influenced our approach. Our core collection may be applied as a testing panel (e.g., to evaluate newly developed genetic markers).

## Conclusions

In this study, a GBS method was used to analyze the genetic diversity and population structure of various European winter wheat cultivars and advanced breeding lines. Because of their quality and regardless of the relatively few markers located in coding sequences, the mapped populations may be used for association mapping, which will serve as the basis for the marker-assisted genomic selection of agronomically important traits. Our results are consistent with those of previous investigations that revealed considerable differences among subgenomes, especially subgenome D, which is characterized by the lowest diversity but the highest LD among the three wheat subgenomes. To the best of our knowledge, this is the first study to identify wheat genes with polymorphisms significantly associated with the year of variety registration. The presented data may be useful for revealing the specific genomic regions that have been targeted during breeding.

The core collection of wheat cultivars representative of the genetic diversity of the currently grown European wheat germplasm described herein may help breeders to increase the genetic diversity of wheat and develop heterotic pools to more efficiently exploit heterosis. It may also serve as a testing panel for developing new marker systems and support the management of wheat genetic resources.

## Methods

### Plant materials

This study was completed with a modern wheat gene pool comprising 277 European varieties that were registered mainly in Germany, Poland, and the United Kingdom during the last 27 years. These varieties were reproduced and delivered by the company Poznań Plant Breeding. Advanced breeding lines were represented by 232 accessions from the ongoing programs of the Plant Breeding Strzelce (STH) and Poznań Plant Breeding (PHR) companies (Table S1). Information regarding cultivars was obtained from the EU database of registered plant varieties.

### Genotyping

For each genotype, DNA was isolated from 15 to 20 bulked 2-week-old seedlings as described by Milligan [86]. The DNA concentration and purity were determined with a NanoDrop spectrophotometer (Thermo Fisher Scientific, Waltham, MA, USA), whereas DNA quality was assessed by 1.5% agarose gel electrophoresis. The DNA was stored at − 20 °C and diluted to a working concentration of 50 ng/μL for the subsequent wheat DArTseq 1.0 genotyping, which was completed by Diversity Arrays Technology (Bruce, Australia).

### Data analysis

The processing of the DArTseq data produced two datasets. First, the SNPs were recorded as codominant markers and were coded as X/Y (i.e., A, C, G, or T) to denote variant alleles at specific loci in homologous chromosomes. The second dataset contained dominant silicoDArT markers resulting from genetic and epigenetic variations at restriction sites during the preparation of libraries. The silicoDArT data represented PAVs and were coded as variants 0 or 1, with 1 representing the homozygous variant present/present or the heterozygous variant present/absent. This enabled the application of the same principles for determining marker parameters, including the variant frequency, minor variant frequency (MVF), and polymorphism information content (PIC), for the SNPs and silicoDArTs.

The BLAST algorithm (version ncbi-blast 2.7.1.) was used to map the trimmed marker sequences to the IWGSC RefSeq (version 1.0) reference genome (Ensembl Plants), with an e-value of 10^− 6^. The linkage disequilibrium (LD) for marker pairs was estimated as *r*^2^ values for fitting a linear regression, with one marker used as the response and another one used as the regressor, and principal component scores used to represent the genetic relatedness of accessions. The hierarchical clustering of markers was based on the LD matrix with a group average (UPGMA) agglomerative method (in R software). Kinship (coancestry) between accessions was estimated with the Dice similarity coefficients computed from marker data. The kinship coefficient matrix was processed via a principal coordinate analysis (PCoA) and used for the hierarchical clustering (complete link method) to visualize the population structure. The Mann-Whitney rank test was used to compare distributions of kinship coefficients between subpopulations. The Ensembl Plants Variant Effect Predictor [87] was applied to annotate SNPs with possible protein translation effects. The association between variety registration year and SNPs was analyzed based on the mixed linear model with the population structure estimated by an eigenanalysis (principal component analysis, PCO) of the matrix of coancestry coefficients estimated from SNP data [88]. The enrichment of Gene Ontology (GO) terms was analyzed with an online tool at Geneontology.org. All analyses not attributed above to R were completed with Genstat 19 [89].

### Marker conversion

Extended sequences adjacent to SNPs selected for conversion to functional markers were retrieved from URGI database [90] and KASP markers were designed with PolyMarker [91].

## Supplementary Information

**Additional file 1.**

**Additional file 2.**

## Data Availability

The genotypic data are available at cropnet.pl/public_data/hybre/hybre1.zip.

## Abbreviations

ANOVA: Analysis of variance
BLAST: Basic Local Alignment Search Tool
CMS: Cytoplasmic male sterility
GBS: Genotyping-by-sequencing
GO: Gene Ontology
GWAS: Genome-wide association study
KASP: Kompetitive allele-specific PCR
LD: Linkage disequilibrium
MVF: Minor variant frequency
PAV: Presence–absence variation
PCO: Principal component analysis
PCoA: Principal coordinate analysis
PCR: Polymerase chain reaction
PHR: Poznań Plant Breeding
PIC: Polymorphism information content
SNP: Single nucleotide polymorphism
SSR: Simple sequence repeat
STH: Plant Breeding Strzelce
UPGMA: Unweighted pair group method with arithmetic mean

## References

[1] FAOSTAT (2019). Production domain. Crops.

[2] Rasheed A, Mujeeb-Kazi A, Ogbonnaya FC, He Z, Rajaram S (2018). Wheat genetic resources in the post-genomics era: promise and challenges. Ann Bot.

[3] Vikram P, Franco J, Burgueño-Ferreira J, Li H, Sehgal D, Saint Pierre C, Ortiz C, Sneller C, Tattaris M, Guzman C, Sansaloni CP, Fuentes-Davila G, Reynolds M, Sonders K, Singh P, Payne T, Wenzl P, Sharma A, Bains NS, Singh GP, Crossa J, Singh S (2016). Unlocking the genetic diversity of creole wheats. Sci Rep.

[4] Li A, Liu D, Yang W, Kishii M, Mao L (2018). Synthetic Hexaploid wheat: yesterday, today, and tomorrow. Engineering.

[5] Marcussen T, Sandve SR, Heier L, Pfeifer M, Kugler KG, Zhan B, Spannagl M, Pfeifer M, Jakobsen KS, BBH W, Steuernagel B, KFX M, Olsen O-A, Sandve SR, Zhan B, Spannagl M, Pfeifer M, Wheat TI, Pfeifer M, Kugler KG, Sandve SR, Zhan B, IWGSC (2014). A chromosome-based draft sequence of the hexaploid bread wheat (*Triticum aestivum*) genome. Science.

[6] Wheat Genome Sequencing Consortium (IWGSC) (2018). Shifting the limits in wheat researchand breeding using a fully annotated reference genome. Science.

[7] Jaiswal SJP, Singh A, Gahatyari NC (2019). Genetic diversity analysis in bread wheat (Triticum aestivum L.em. Thell.) for yield and physiological traits. Int J Curr Microbiol Appl Sci.

[8] Whitford R, Fleury D, Reif JC, Garcia M, Okada T, Korzun V, Langridge P (2013). Hybrid breeding in wheat: technologies to improve hybrid wheat seed production. J Exp Bot.

[9] Tucker EJ, Baumann U, Kouidri A, Suchecki R, Baes M, Garcia M, Okada T, Dong C, Wu Y, Sandhu A, Singh M, Langridge P, Wolters P, Albertsen MC, Cigan AM, Whitford R (2017). Molecular identification of the wheat male fertility gene Ms1 and its prospects for hybrid breeding. Nat Commun.

[10] Longin CFH (2013). Hybrid wheat: quantitative genetic parameters and consequences for the design of breeding programs. Theor Appl Genet.

[11] Muhleisen J, Piepho HP, Maurer HP, Longin CF, Reif JC (2014). Yield stability of hybrids versus lines in wheat, barley, and triticale. Theor Appl Genet.

[12] Ni F, Qi J, Hao Q, Lyu B, Luo MC, Wang Y, Chen F, Wang S, Zhang C, Epstein L, Zhao X, Wang H, Zhang X, Chen C, Sun L, Fu D (2017). Wheat Ms2 encodes for an orphan protein that confers male sterility in grass species. Nat Commun.

[13] Longin C (2012). Hybrid breeding in autogamous cereals. Theor Appl Genet.

[14] Xia C, Zhang L, Zou C, Gu Y, Duan J, Zhao G, Wu J, Liu Y, Fang X, Gao L, Jiao Y, Sun J, Pan Y, Liu X, Jia J, Kong X (2017). A TRIM insertion in the promoter of Ms2 causes male sterility in wheat. Nat Commun.

[15] Bohra A, Jha UC, Adhimoolam P (2016). Cytoplasmic male sterility (CMS) in hybrid breeding in field crops. Plant Cell Rep.

[16] Bohn M, Friedrich UH, Melchinger AE (1999). Genetic similarities among winter wheat cultivars determined on the basis of RFLPs, AFLPs and SSRs and their use for predicting progeny variance. Crop Sci.

[17] Prasad M, Varshney RK, Roy JK, Balyan HS, Gupta PK (2000). The use of microsatellites for detecting DNA polymorphism, genotype identification and genetic diversity in wheat. Theor Appl Genet.

[18] Landjeva S, Korzun V, Ganeva G (2006). Evaluation of genetic diversity among Bulgarian winter wheat (Triticum aestivum L.) varieties during the period 1925–2003 using microsatellites. Genet Resour Crop Ev.

[19] Prasad B, Babar MA, Xu XY, Bai GH, Klatt AR (2009). Genetic diversity in the U.S. hard red winter wheat cultivars as revealed by microsatellite markers. Crop Pasture Sci.

[20] Zhuang PP, Ren QC, Li W, Chen GY (2011). Genetic diversity of Persian wheat (Triticum turgidum ssp. carthlicum) accessions by EST-SSR markers. Am J Biochem Mol Biol.

[21] Arora A, Kundu S, Dilbaghi N, Sharma I, Tiwari R (2014). Population structure and genetic diversity among Indian wheat varieties using microsatellite (SSR) markers. Aust J Crop Sci.

[22] Wingen LU, West C, Waite ML, Collier S, Orford S, Goram R, Yang CY, King J, Allen AM, Burridge A, Edwards KJ, Griffiths S (2017). Wheat landrace genome diversity. Genetics.

[23] Allen AM, Barker GL, Berry ST, Coghill JA, Gwilliam R, Kirby S, Robinson P, Brenchley RC, D'Amore R, McKenzie N, Waite D, Hall A, Bevan M, Hall N, Edwards KJ (2011). Transcript- specific, single-nucleotide polymorphism discovery and linkage analysis in hexaploid bread wheat (Triticum aestivum L.). Plant Biotechnol J.

[24] Jia M, Guan J, Zhai Z, Geng S, Zhang X, Mao L, Li A (2018). Wheat functional genomics in the era of next generation sequencing: an update. Crop J.

[25] Sansaloni C, Petroli C, Jaccoud D (2011). Diversity arrays technology (DArT) and next-generation sequencing combined: genome-wide, high throughput, highly informative genotyping for molecular breeding of eucalyptus. BioMed Cent.

[26] Poland JA, Brown PJ, Sorrells ME, Jannink JL (2012). Development of high-density genetic maps for barley and wheat using a novel two-enzyme genotyping-by-sequencing approach. PLoS One.

[27] Courtois B, Audebert A, Dardou A (2013). Genome-wide association mapping of root traits in a japonica rice panel. PLoS One.

[28] Sehgal D, Vikram P, Sansaloni CP (2015). Exploring and mobilizing the gene bank biodiversity for wheat improvement. PLoS One.

[29] Cavanagh CR, Chao S, Wang S, Huang BE, Stephen S, Kiani S, Forrest K, Saintenac C, Brown-Guedira GL, Akhunova A, See D, Bai G, Pumphrey M, Tomar L, Wong D, Kong S, Reynolds M, Lopez da Silva M, Bockelman H, Talbert L, Anderson JA, Dreisigacker S, Baenziger S, Carter A, Korzun V, Morrell PL, Dubcovsky J, Morell MK, Sorrells ME, Hayden MJ, Akhunov E (2013). Genome-wide comparative diversity uncovers multiple targets of selection for improvement in hexaploid wheat landraces and cultivars. PNAS.

[30] Jordan KW, Wang S, Lun Y (2015). A haplotype map of allohexaploid wheat reveals distinct patterns of selection on homoeologous genomes. Genome Biol.

[31] Riaz A, Hathorn A, Dinglasan E (2016). Into the vault of the Vavilov wheats: old diversity for new alleles. Genet Resour Crop Ev.

[32] Shi F, Tibbits J, Pasam RK (2017). Exome sequence genotype imputation in globally diverse hexaploid wheat accessions. Theor Appl Genet.

[33] Ren J, Sun D, Chen L (2013). Genetic diversity revealed by single nucleotide polymorphism markers in a worldwide germplasm collection of durum wheat. Int J Mol Sci.

[34] Scherlosky A, Marchioro VS, de Assis FF, Braccini AL, Schuster I (2018). Genetic variability of Brazilian wheat germplasm obtained by high-density SNP genotyping. Crop Breed Appl Biotech.

[35] Tadesse W, Ogbonnaya FC, Jighly A (2015). Genome-Wide Association Mapping of yield and grain quality traits in winter wheat genotypes. PLoS One.

[36] Breseghello F, Sorrells ME (2006). Association mapping of kernel size and milling quality in wheat (Triticum aestivum L.) cultivars. Genetics.

[37] Massman J, Cooper B, Horsley R (2006). Genome-wide association mapping of Fusarium head blight resistance in contemporary barley breeding germplasm. Mol Breed.

[38] Joukhadar R, El-Bouhssini M, Jighly A, Ogbonnaya FC (2013). Genome-wide association mapping for five major pest resistances in wheat. Mol Breed.

[39] Frankel OH, Arber W, Llimensee K, Peacock WJ, Starlinger P (1984). Genetic perspectives of germplasm conservation. Genetic Manipulation: Impact on Man and Society.

[40] Brown AHD (1989). Core collections: a practical approach to genetic resources management. Genome.

[41] Brown AHD, Hodgkin T, Brown HD, van Hintum TL, Morales EV (1995). The core collection at the crossroads. Core collections of plant genetic resources.

[42] Odong TL, Jansen J, van Eeuwijk FA, van Hintum TJL (2013). Quality of core collections for effective utilisation of genetic resources review, discussion and interpretation. Theor Appl Genet.

[43] Research Centre for Cultivar Testing. Varieties comparison. https://coboru.gov.pl/PDO/porownanieodmian.aspx. Accessed 23 June 2019.

[44] Poland J, Endelman J, Dawson J, Rutkoski J, Wu S, Manes Y (2012). Genomic selection in wheat breeding using genotyping-by-sequencing. Plant Genome.

[45] Saintenac C, Jiang D, Wang S, Akhunov E (2013). Sequence-based mapping of the polyploid wheat genome. G3 genes, genomes. Genet.

[46] Wang SC, Wong DB, Forrest K, Allen A, Chao SM, Huang BE, Mac-caferri M, Salvi S, Milner SG, Cattivelli L, Mastrangelo AM, Whan A, Stephen S, Barker G, Wieseke R, Plieske J, Lillemo M, Mather D, Appels R, Dolferus R, Brown-Guedira G, Korol A, Akhunova AR, Feuillet C, Salse J, Morgante M, Pozniak C, Luo MC, Dvorak J, Morell M, Dubcovsky J, Ganal M, Tuberosa R, Lawley C, Mikoulitch I, Cavanagh C, Edwards KJ, Hayden M, Akhunov E, Sequencing IWG (2014). Characterization of polyploid wheat genomic diversity using a high-density 90,000 single nucleotide polymorphism array. Plant Biotechnol J.

[47] Burridge AJ, Winfield MO, Allen AM, Wilkinson PA, Barker GLA, Coghill J, Waterfall C, Edwards KJ, Bhalla PL, Singh MB (2017). High-density SNP genotyping Array for Hexaploid wheat and its relatives. Wheat biotechnology: methods and protocols.

[48] Rimbert H, Darrier B, Navarro J, Kitt J, Choulet F, Leveugle M, Duarte J, Riviere N, Eversole K, Le Gouis J, Davassi A, Balfourier F, Le Paslier M-C, Lie Berard A, Brunel D, Feuillet C, Poncet C, Sourdille P, Paux E (2018). High throughput SNP discovery and genotyping in hexaploid wheat. PLoS One.

[49] Winfield MO, Allen AM, Burridge AJ, Barker GLA, Benbow HR, Wilkinson PA, Coghill J, Waterfall C, Davassi A, Scopes G, Pirani A, Webster T, Brew F, Bloor C, King J, West C, Griffiths S, King I, Bentley AR, Edwards KJ (2016). High-density SNP genotyping array for hexaploid wheat and its secondary and tertiary gene pool. Plant Biotechnol J.

[50] Zhou S, Zhang J, Che Y, Liu W, Lu Y, Yang X, Li X, Jia J, Liu X, Li L (2017). Construction of Agropyron Gaertn. Genetic linkage maps using a wheat 660K SNP array reveals a homoeologous relationship with the wheat genome. Plant Biotechnol J.

[51] Balfourier F, Bouchet S, Robert S, DeOliveira R, Rimbert H, Kitt J, Choulet F, Paux E (2019). Worldwide phylogeography and history of wheat genetic diversity. Sci Adv.

[52] Shaked H, Kashkush K, Ozkan H, Feldman M, Levy AA (2001). Sequence elimination and cytosine methylation are rapid and reproducible responses of the genome to wide hybridization and allopolyploidy in wheat. Plant Cell.

[53] Yu J, Buckler ES (2006). Genetic association mapping and genome organization of maize. Curr Opin Biotechnol.

[54] Sukumaran S, Dreisigacker S, Lopes M, Chavez P, Reynolds MP (2015). Genome-wide association study for grain yield and related traits in an elite spring wheat population grown in temperate irrigated environments. Theor Appl Genet.

[55] Liu J, Rasheed A, He Z, Imtiaz M, Arif A (2019). Genome-wide variation patterns between landraces and cultivars uncover divergent selection during modern wheat breeding. Theor Appl Genet.

[56] Alipour H, Bihamta MR, Mohammadi V, Peyghambari SA, Bai G, Zhang G (2017). Genotyping-by-sequencing (GBS) revealed molecular genetic diversity of Iranian wheat landraces and cultivars. Front Plant Sci.

[57] Eltaher S, Sallam A, Belamkar V, Emara HA, Nower AA, Salem KFM, Poland J, Baenziger PS (2018). Genetic diversity and population structure of F3:6 Nebraska winter wheat genotypes using genotyping-by-sequencing. Front Genet.

[58] Akhunov ED, Akhunova AR, Anderson OD, Anderson JA, Blake N, Clegg MT, Coleman-Derr D, Conley EJ, Crossman CC, Deal KR, Dubcovsky J, Gill BS, Gu YQ, Hadam J, Heo H, Huo N, Lazo GR, Luo MC, Ma YQ, Matthews DE, McGuire PE, Morrell PL, Qualset CO, Renfro J, Tabanao D, Talbert LE, Tian C, Toleno DM, Warburton ML, You FM (2010). Nucleotide diversity maps reveal variation in diversity among wheat genomes and chromosomes. BMC Genomics.

[59] Würschum T (2013). Population structure, genetic diversity and linkage disequilibrium in elite winter wheat assessed with SNP and SSR markers. Theor Appl Genet.

[60] Chao S, Dubcovsky J, Dvorak J, Luo M-C, Baenziger SP, Matnyazov R, Clark DR, Talbert LE, Anderson JA, Dreisigacker S, Glover K, Chen J, Campbell K, Bruckner PL, Rudd JC, Haley S, Carver BF, Perry S, Sorrells ME, Akhunov ED (2010). Population- and genome-specific patterns of linkage disequilibrium and SNP variation in spring and winter wheat (i L.). BMC Genomics.

[61] Mirzaghaderi G, Mason AS (2019). Broadening the bread wheat D genome. Theor Appl Genet.

[62] Chao S, Zhang W, Akhunov E, Sherman J, Ma Y, Luo MC, Dubcovsky J (2009). Analysis of gene-derived SNP marker polymorphism in US wheat (Triticum aestivum L.) cultivars. Mol Breed.

[63] Lopes M, Dreisigacker S, Peña R, Sukumaran S, Reynolds M (2014). Genetic characterization of the wheat association mapping initiative (WAMI) panel for dissection of complex traits in spring wheat. Theor Appl Genet.

[64] Liu J, He Z, Rasheed A (2017). Genome-wide association mapping of black point reaction in common wheat (*Triticum aestivum* L.). BMC Plant Biol.

[65] Mir RR, Kumar J, Balyan HS, Gupta PK (2012). A study of genetic diversity among Indian bread wheat (Triticum aestivum L.) cultivars released during last 100 years. Genet Resour Crop Evol.

[66] Bhatta M, Morgounov A, Belamkar V (2018). Unlocking the novel genetic diversity and population structure of synthetic Hexaploid wheat. BMC Genomics.

[67] Roncallo PF, Beaufort V, Larsen AO, Dreisigacker S, Echenique V (2019). Genetic diversity and linkage disequilibrium using SNP (KASP) and AFLP markers in a worldwide durum wheat (*Triticum turgidum* L. var durum) collection. PLoS One.

[68] Joukhadar R, Daetwyler HD, Bansal U, Gendall AR, Hayden MJ (2017). Genetic diversity, Population Structure and Ancestral Origin of Australian Wheat. Front Plant Sci.

[69] Rufo R, Alvaro F, Royo C, Soriano JM (2019). From landraces to improved cultivars: assessment of genetic diversity and population structure of Mediterranean wheat using SNP markers. PLoS One.

[70] Choulet F, Alberti A, Theil S, Glover N, Barbe V, Daron J (2014). Structural and functional partitioning of bread wheat chromosome 3B. Science.

[71] Glover N, Daron J, Pingault L, Vandepoele K, Paux E, Feuillet C (2015). Small-scale gene duplications played a major role in the recent evolution of wheat chromosome 3B. Genome Biol.

[72] Montenegro JD, Golicz AA, Bayer PE, Hurgobin B, Lee H, Chan CKK, Visendi P, Lai K, Dolezel J, Batley J, Edwards D (2017). The pangenome of hexaploid bread wheat. Plant J.

[73] Brenchley R, Spannagl M, Pfeifer M, Barker GLA, D’Amore R, Allen AM, McKenzie N, Kramer M, Kerhornou A, Bolser D, Kay S, Waite D, Trick M, Bancroft I, Gu Y, Huo N, Luo MC, Sehgal S, Gill B, Kianian S, Anderson O, Kersey P, Dvorak J, McCombie WR, Hall A, Mayer KFX, Edwards KJ, Bevan MW, Hall N (2012). Analysis of the bread wheat genome using whole-genome shotgun sequencing. Nature.

[74] Feldman M, Levy AA (2005). Allopolyploidy - a shaping force in the evolution of wheat genomes. Cytogenet Genome Res.

[75] Kumar D, Chhokar V, Sheoran S (2020). Characterization of genetic diversity and population structure in wheat using array based SNP markers. Mol Biol Rep.

[76] Charlesworth D, Willis JH (2009). The genetics of inbreeding depression. Nat Rev Genet.

[77] Uniprot Database. https://www.uniprot.org. Accessed 12 Mar 2020.

[78] Hedden P, Sponsel VA (2015). Century of gibberellin research. J Plant Growth Regul.

[79] Skubacz A, Daszkowska-Golec A. Seed dormancy: the complex process regulated by Abscisic acid, gibberellins, and other Phytohormones that makes seed germination work. In: El-Esawi M, editor. Phytohormones - signaling mechanisms and crosstalk in plant development and stress responses. London: InTech; 2017.

[80] Peng J, Richards DE, Hartley NM, Murphy GP, Devos KM (1999). ‘Green revolution’ genes encode mutant gibberellin response modulators. Nature.

[81] Joshi S, Choukimath A, Isenegger D, Panozzo J, Spangenberg G, Kant S (2019). Improved wheat growth and yield by delayed leaf senescence using developmentally regulated expression of a Cytokinin biosynthesis gene. Front Plant Sci.

[82] Mackay I, Horwell A, Garner J, White J, McKee J, Philpott H (2011). Reanalysis of the historical series of UK variety trials to quantify the contributions of genetic and environmental factors to trends and variability in yield over time. Theor Appl Genet.

[83] Research Centre for Cultivar Testing. http://www.coboru.pl. Accessed 23 June 2020.

[84] Targońska M, Bolibok-Bragoszewska H, Rakoczy-Trojanowska M (2016). Assessment of genetic diversity in Secale cereale based on SSR markers. Plant Mol Biol Rep.

[85] van Hintum TJL, Johnson RC, Hodgkin T (1999). The general methodology for creating a core collection. Core collections for today and tomorrow.

[86] Milligan BG, Hoelzel AR (1992). Plant DNA isolation. Molecular analysis of populations: a practical approach.

[87] McLaren W, Gil L, Hunt SE, Riat HS, Ritchie GR, Thormann A, Flicek P, Cunningham F (2016). The Ensembl Variant Effect Predictor. Genome Biol.

[88] Malosetti M, Ribaut JM, van Eeuwijk FA (2013). The statistical analysis of multi-environment data: modeling genotype-by-environment interaction and its genetic basis. Front Physiol.

[89] VSN International (2017). Genstat for Windows.

[90] Alaux M, Rogers J, Letellier T, Flores R, Alfama F, Pommier C, Mohellibi N, Durand S, Kimmel E, Michotey C, Guerche C, Loaec M, Lainé M, Steinbach D, Choulet F, Rimbert H, Leroy P, Guilhot N, Salse J, Feuillet C, Paux E, Eversole K, Adam-Blondon AF, Quesneville H, International Wheat Genome Sequencing Consortium (2018). Linking the International Wheat Genome Sequencing Consortium bread wheat reference genome sequence to wheat genetic and phenomic data. Genome Biol.

[91] Ramirez-Gonzalez RH, Uauy C, Caccamo M (2015). PolyMarker: a fast polyploid primer design pipeline. Bioinformatics.

[92] Rosyara U, Kishii M, Payne T (2019). Genetic contribution of synthetic Hexaploid wheat to CIMMYT’s spring bread wheat breeding Germplasm. Sci Rep.

