# Supplementary material for: Evaluation of genetic structure in European wheat cultivars and advanced breeding lines using high-density genotyping-by-sequencing approach
Source: BMC Genomics. 2021 Jan 28;22:81. doi: 10.1186/s12864-020-07351-x (PMC7842024; doi:10.1186/s12864-020-07351-x)
Supplement: Supplementary file 2 — Additional file 2. [file 12864_2020_7351_MOESM2_ESM.docx]

| 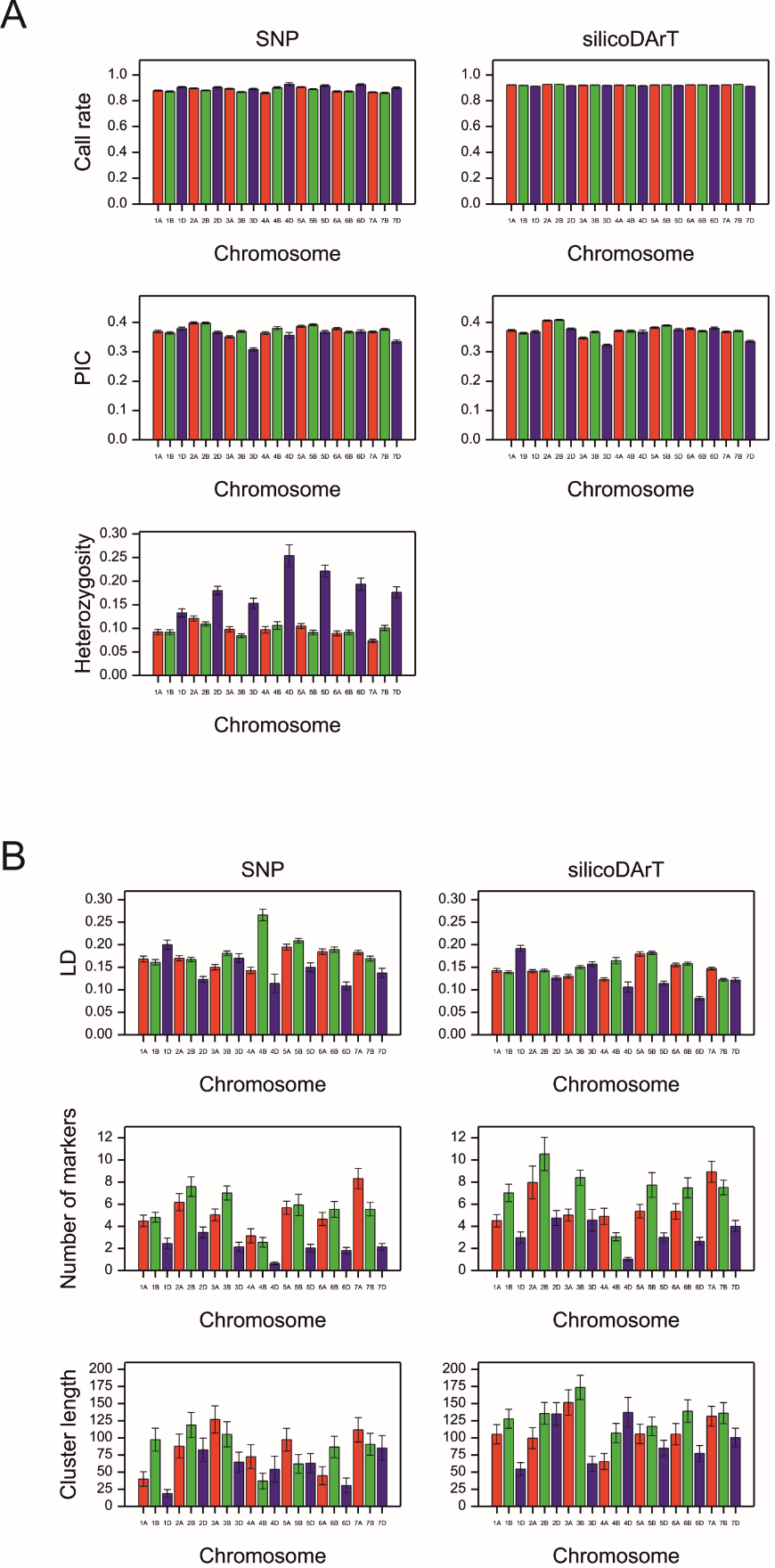 | |  | |
| --- | --- | --- | --- |
|  |  |  |  |
|  | |  | |
| Fig S1. A. Characteristics of SNP and silicoDArT marker datasets by chromosomes. B. Characteristics of LD and of clusters of markers selected by LD analysis. Mean values, S.E.M. computed over markers in each chromosome. | | | |

|  |
| --- |
|  |
|  |
| 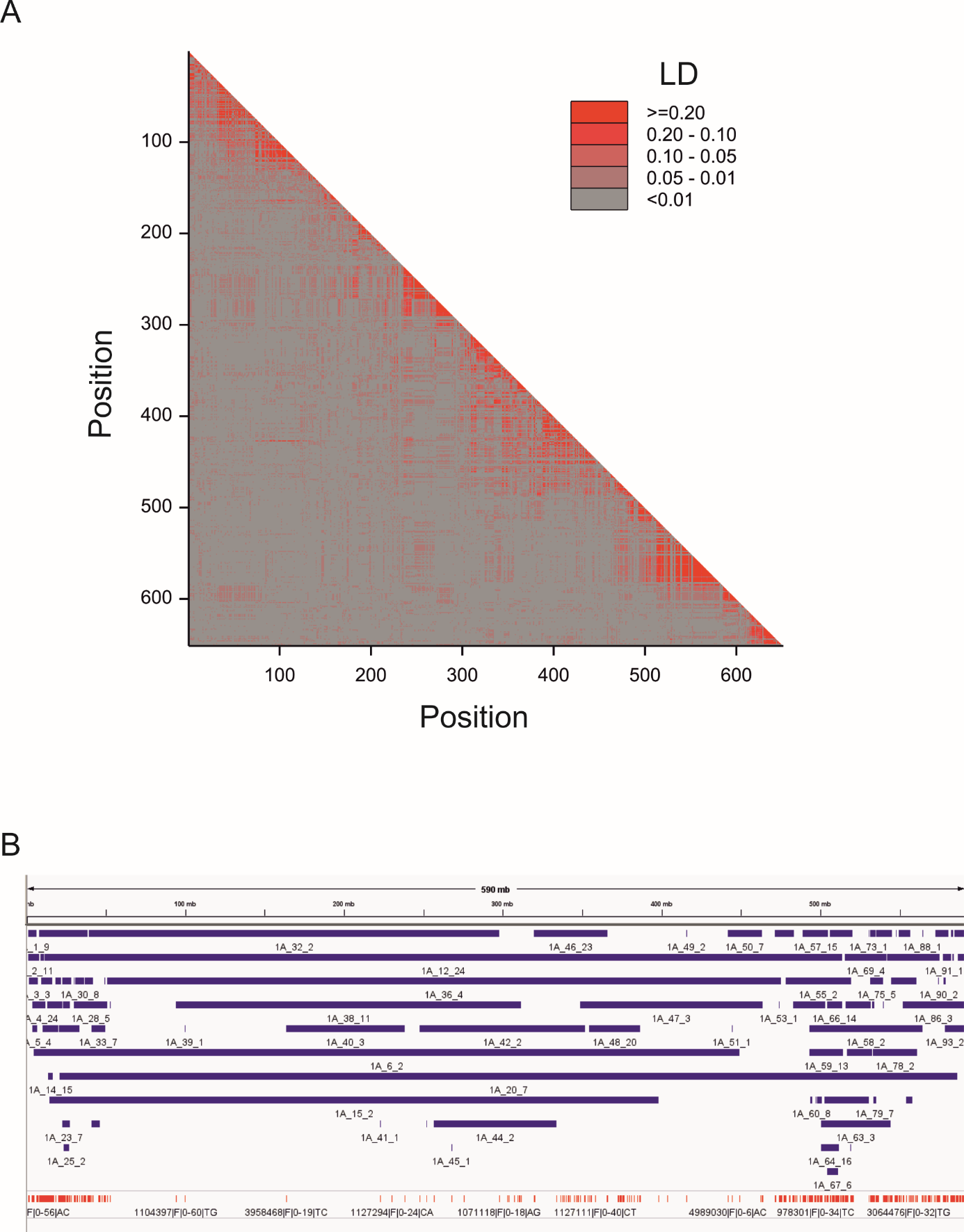 |
| Fig. S2. A. LD structure in chromosme 1A . B. LD clusters of SNP markers in chromosome 1A. |


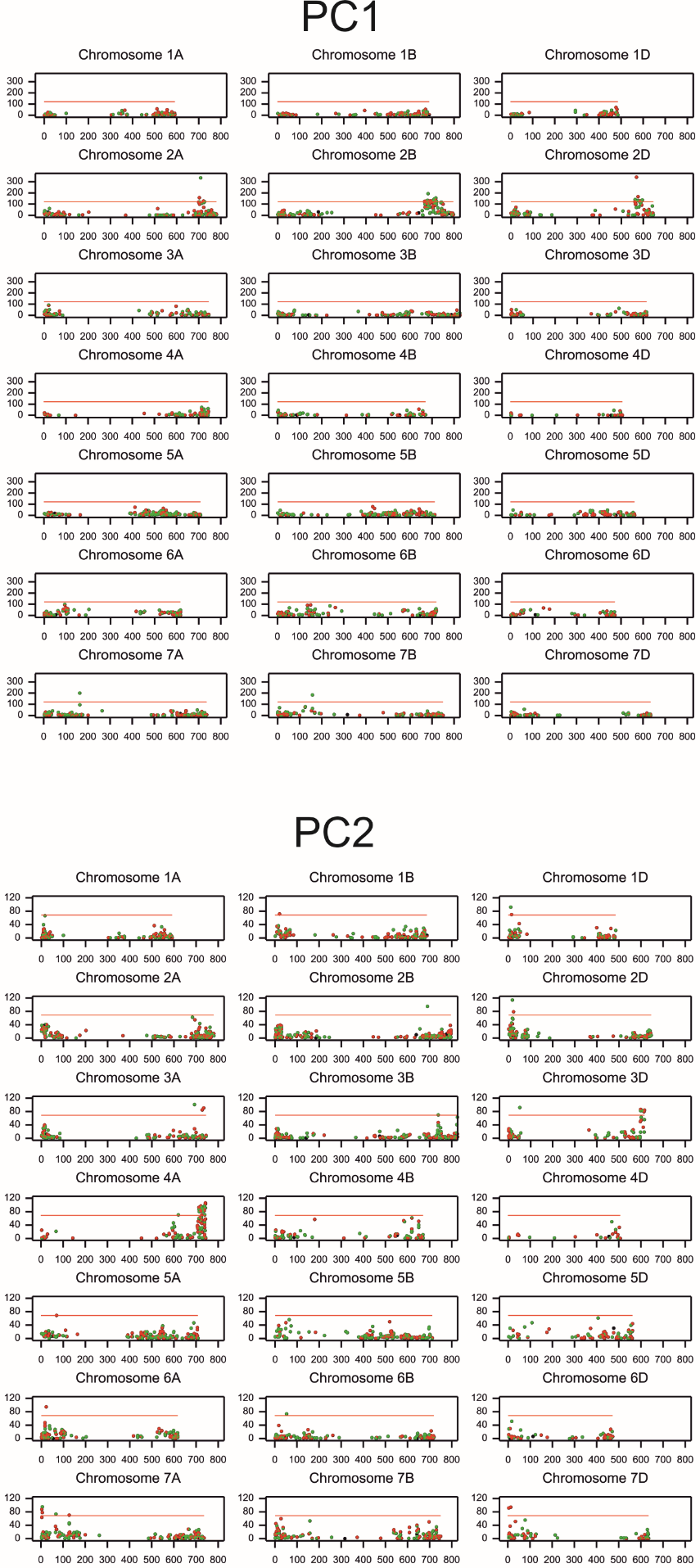


Fig. S3. Localization of SNPs with HIGH (black), LOW (red) or MODERATE (green) coding effects and polymorphism correlated to variability represented by PC1 and PC2 of population structure in Fig. 6. Red line marks the 99th percentile of the distribution of F statistic values (121.2 for PC1, 68.7 for PC2).


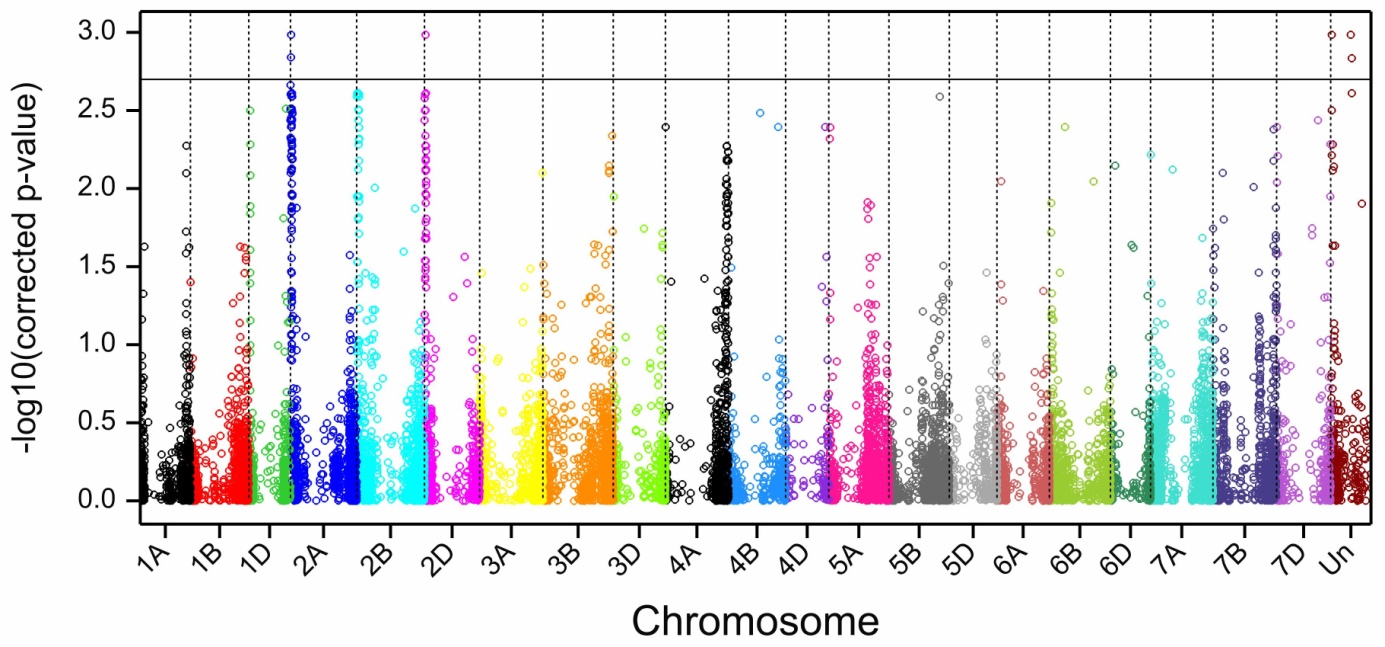


Fig. S4. Manhattan plot illustrating results of association analysis for trait "registration year" using 13499 SNP markers in 21 chromosomes of wheat and chromosome Un. Six SNPs with largest significance score (corrected p-value < 0.002) in chromosomes 2A, 2D and Un are characterized in details in Tables 2 and 3.

| 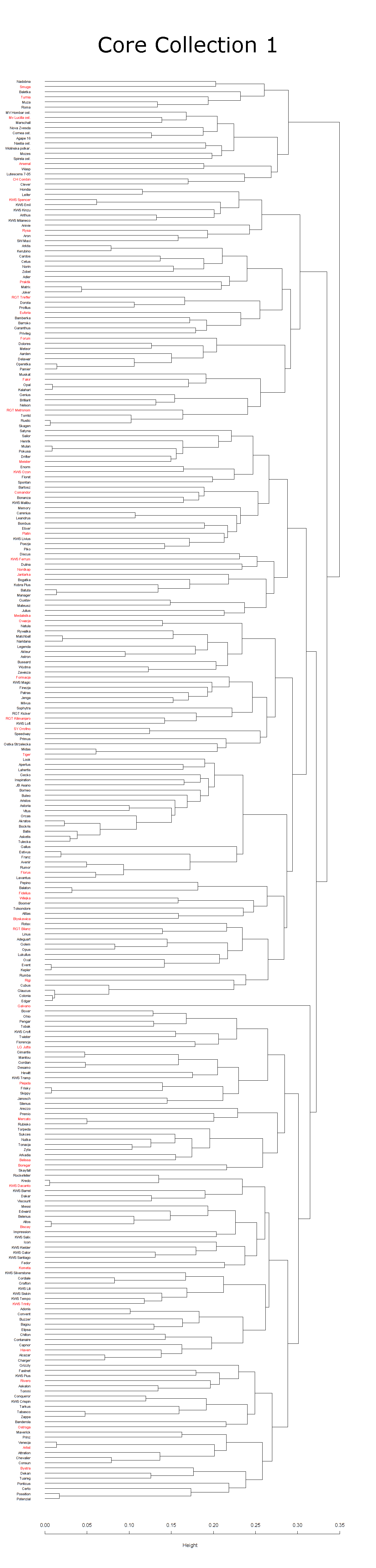 | 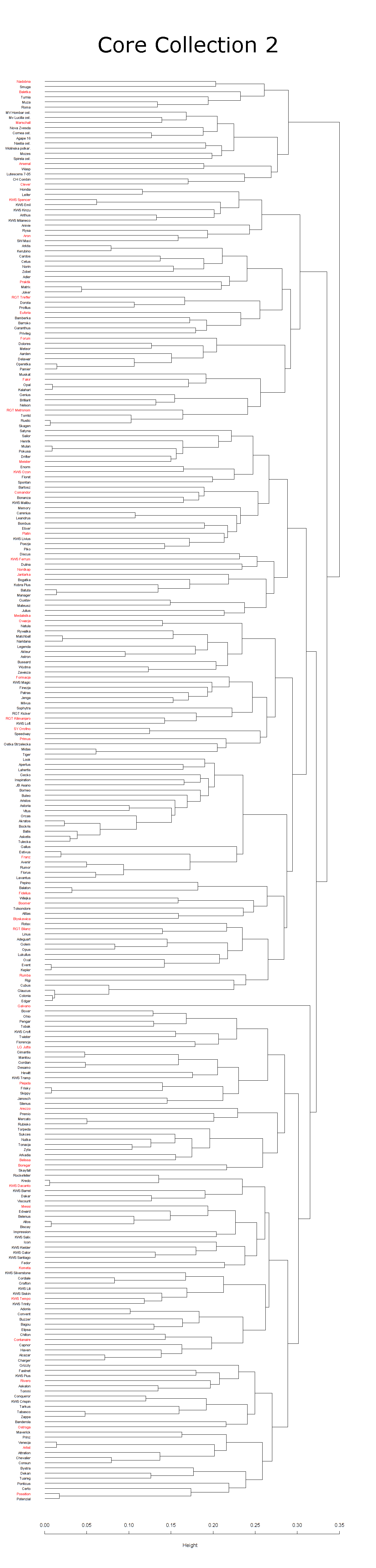 |
| --- | --- |
| Fig. S5. Dendrograms of whole variety collections. Red color indicates accessions belonging to core collections 1 and 2. | |


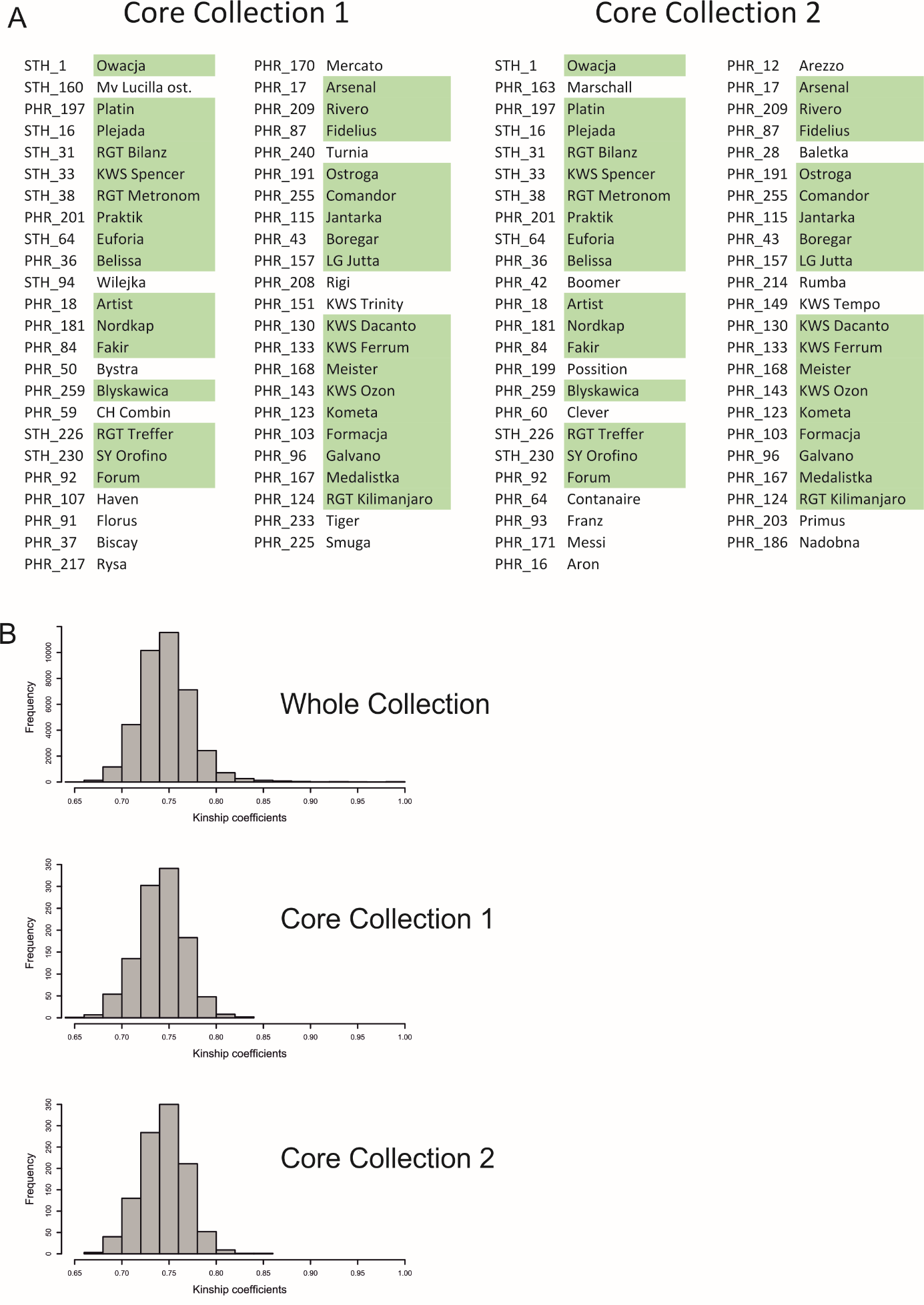


Figure S6. Core collections. A. Lists of varieties constituting core collections 1 and 2. Varieties belonging to both core collections are marked in green. B. Histograms of kinship coefficients for the whole collection and core collections 1 and 2.
